# Achievements and Challenges in Therapy and Vaccines Development of Viral Hemorrhagic Fevers: An Up-to-Date Review

**DOI:** 10.3390/pharmaceutics18040426

**Published:** 2026-03-30

**Authors:** Dan Lupascu, Andreea-Teodora Iacob, Maria Apotrosoaei, Ioana-Mirela Vasincu, Florentina-Geanina Lupascu, Oana-Maria Chirliu, Bianca-Stefania Profire, Roxana-Georgiana Tauser, Lenuta Profire

**Affiliations:** 1 Department of Pharmaceutical and Therapeutical Chemistry, Faculty of Pharmacy, Grigore T. Popa University of Medicine and Pharmacy Iasi, 16 Universitatii Street, 700115 Iasi, Romania; dan.lupascu@umfiasi.ro (D.L.); apotrosoaei.maria@umfiasi.ro (M.A.); ioana-mirela.vasincu@umfiasi.ro (I.-M.V.); florentina-geanina.lupascu@umfiasi.ro (F.-G.L.); oana-maria.ionescu@umfiasi.ro (O.-M.C.); roxana.tauser@umfiasi.ro (R.-G.T.); lenuta.profire@umfiasi.ro (L.P.); 2 Department of Internal Medicine, Faculty of Medicine, Grigore T. Popa University of Medicine and Pharmacy Iasi, 16 Universitatii Street, 700115 Iasi, Romania; bianca-stefania.profire@umfiasi.ro

**Keywords:** viral hemorrhagic fevers, Ebola, Marburg, dengue, yellow fever, antiviral therapy, monoclonal antibodies, immunoprophylaxis, vaccines

## Abstract

Viral hemorrhagic fevers (VHFs) comprise a heterogeneous group of severe infectious diseases that continue to represent a major global health concern. Although many VHFs remain endemic to regions of Africa, Asia, and the Americas, their wide geographic distribution, together with increasing international travel and global trade, facilitates the importation of cases into non-endemic areas and raises the risk of secondary transmission under favorable ecological and epidemiological conditions. These infections are frequently associated with high case-fatality rates and impose a substantial social and economic burden, including pressure on healthcare systems, disruption of essential services, and long-term physical and psychological sequelae among survivors. Despite notable advances in recent years, therapeutic options for VHFs remain limited. Supportive care continues to represent the cornerstone of clinical management for most infections, while pathogen-targeted therapies are available only for a restricted number of diseases. Monoclonal antibody-based therapies have achieved the most significant regulatory success to date, particularly for Ebola virus disease. In parallel, several small-molecule antivirals have been investigated in preclinical and clinical settings, including during outbreak responses, although inconsistent efficacy and safety concerns have limited widespread approval. Vaccine development has progressed further, with licensed vaccines available for selected VHFs, including Ebola, yellow fever, and dengue, and multiple candidates based on diverse technological platforms advancing through clinical evaluation. In addition to summarizing current therapeutic and vaccine strategies, this review highlights pharmaceutical development considerations relevant to biologic therapeutics and selected vaccine platforms, including formulation stability, pharmacokinetic behavior, delivery routes, storage requirements, and logistical constraints affecting deployment during outbreak responses. Using a comparative cross-pathogen framework, the review synthesizes recent literature to identify translational gaps, regulatory challenges, and future priorities for the development of safer and more effective medical countermeasures against VHFs.

## 1. Introduction

Viral hemorrhagic fevers (VHFs) are a general term for a group of severe diseases caused by RNA viruses belonging to the families *Arenaviridae*, *Filoviridae*, *Flaviviridae*, *Hantaviridae*, *Nairoviridae*, *Paramyxoviridae* and *Phenuiviridae*. Detailed information about the viruses responsible for VHFs, including their species, geographic distribution, reservoir hosts, and incubation periods, is provided in Supplementary Table S1 [1,2].

The viruses responsible for VHFs are typically classified as Biosafety Level 4 (BSL-4) pathogens, requiring specialized containment facilities and strict safety protocols [3]. These infections—including Ebola, dengue, and Marburg virus disease (MVD)—are characterized by fever, systemic toxicity, and, in severe cases, hemorrhagic manifestations associated with high case-fatality rates. Collectively, VHFs account for an estimated 51–101 million cases and approximately 67,000 deaths annually worldwide [4,5]. As summarized in Table S1, transmission occurs via direct contact with infected animals or through hematophagous arthropod vectors, enabling both zoonotic spillover and, in some cases, human-to-human transmission. VHFs are endemic in regions of Africa, South America, and Asia, and their geographic spread may be facilitated by global mobility and trade [6]. Historical and contemporary examples, including the spread of yellow fever and the dissemination of dengue and Ebola viruses, illustrate their capacity to expand beyond original endemic zones [6,7].

Current therapeutic options for VHFs remain limited and are largely based on supportive care. Licensed pathogen-targeted therapies are essentially restricted to Ebola virus disease, for which two monoclonal antibody (mAb) products, Inmazeb and Ebanga, have received regulatory approval. For most other VHFs, no approved specific antiviral therapy is available, and management continues to rely primarily on supportive measures, with certain agents, such as ribavirin, used off-label in selected clinical settings. In contrast, vaccine development has shown comparatively greater progress, with licensed vaccines available for a limited number of VHFs and multiple candidates undergoing clinical evaluation. Much of the evidence supporting emerging therapeutic and vaccine candidates derives from preclinical studies, particularly non-human primate (NHP) models, which represent the most informative experimental system for filovirus research; however, translating these findings into consistent clinical benefit in humans remains challenging due to differences in disease progression, treatment timing, and the logistical constraints of conducting controlled trials during outbreaks.

Unlike previous pathogen-specific reviews, the present work adopts a comparative cross-pathogen framework integrating therapeutic and vaccine development strategies across multiple VHFs. In this context, the review provides an updated and comprehensive analysis of current therapeutic agents and vaccines developed for major VHFs, including Ebola virus disease, Sudan virus disease, Marburg virus disease, dengue, and yellow fever. The primary objective is to critically evaluate both approved and investigational countermeasures in relation to viral structure, pathogenesis, and epidemiology, while also emphasizing pharmaceutical development aspects relevant to biologic therapeutics and antiviral agents. Particular attention is given to formulation strategies, pharmacokinetic characteristics, delivery routes, and stability considerations that influence the practical development, distribution, and deployment of therapeutics and vaccines during outbreak responses. By integrating virological, clinical, and pharmaceutical perspectives and synthesizing the most recent specialized literature, this review aims to identify current achievements, remaining challenges, and future priorities for the development of safer, more effective, and pharmaceutically optimized medical countermeasures against viral hemorrhagic fevers.

## 2. Literature Search Strategy

To ensure comprehensive coverage of the available evidence, a structured literature search was conducted using the electronic databases PubMed, Scopus, and Web of Science. This review was conducted as a narrative literature review, focusing on the synthesis of current evidence regarding therapeutic agents, vaccine development, and pharmaceutical considerations for major viral hemorrhagic fevers. The search included articles published from January 2000 to January 2026, with particular emphasis on studies published in the last decade. Keywords and combinations of keywords included “viral haemorrhagic fever”, “Ebola virus”, “Marburg virus”, “Sudan virus”, “dengue”, “yellow fever”, “antiviral therapy”, “monoclonal antibodies”, “vaccines”, “drug development”, and “clinical trials”.

Peer-reviewed articles published in English that addressed the virology, epidemiology, therapeutic strategies, vaccine development, and pharmaceutical aspects of viral hemorrhagic fevers were considered for inclusion. Both experimental and clinical studies, as well as relevant review articles, were evaluated to provide a comprehensive overview of current knowledge. Reports lacking sufficient methodological detail, conference abstracts without full publications, and duplicate records were excluded.

In addition to database searches, the reference lists of selected articles were manually screened to identify additional relevant publications. Regulatory documents and reports from international health organizations were also consulted to obtain updated information on approved therapeutics, vaccine candidates, and outbreak-related developments.

## 3. Ebola Disease

Ebola disease (EBOD), also known as Ebola hemorrhagic fever, is a severe infection affecting humans and non-human primates (NHPs), caused by several species of *Orthoebolavirus* (*O. zairense*, *O. sudanense*, *O. bundibugyoense* and *O. taiense*) within the *Filoviridae* family. Among these, Zaire Ebola virus (EBOV), a single-stranded negative-sense RNA virus, is the most virulent, with case fatality rates reaching up to 90% in untreated patients. Transmission occurs primarily through direct contact with infected bodily fluids or contaminated materials, as well as exposure to infected animals, particularly fruit bats. Viral RNA has also been detected in breast milk, vaginal secretions, and semen, supporting the potential for sexual transmission [8,9].

Ebola virus disease (EVD) is endemic in parts of Central and West Africa, with documented spread across national borders during outbreaks [9,10]. First identified in 1976 in Zaire (now the Democratic Republic of the Congo, DRC), EVD has caused multiple outbreaks, including the large West African epidemic (2014–2016), which resulted in over 28,000 cases and more than 11,000 deaths [10,11,12]. Subsequent outbreaks have continued to occur, particularly in the DRC and neighboring regions [13,14]. A chronological overview of major outbreaks is provided in Supplementary Table S2 [10,15,16].

In addition to acute disease manifestations, EBOV may persist in immune-privileged anatomical sites following apparent clinical recovery. Viral RNA and, in some cases, infectious virus have been detected in compartments such as the central nervous system, ocular tissues, and testes for prolonged periods. This persistence has been associated with rare cases of relapse or delayed disease manifestations. Although true reinfection appears to be rare, most recurrence events are attributed to long-term viral persistence in these anatomical reservoirs. The underlying mechanisms remain incompletely understood and have important implications for survivor management and outbreak control [17,18,19].

Clinically, EVD presents as an acute febrile illness with nonspecific early symptoms, including fever, headache, myalgia, and weakness, followed by gastrointestinal manifestations such as vomiting and diarrhea, often leading to severe dehydration [20,21]. As the disease progresses, neurological symptoms and systemic involvement may occur [20,22].

In advanced stages, hemorrhagic complications, circulatory instability, and multi-organ dysfunction develop, frequently resulting in metabolic disturbances and organ failure [12,20,21].

Survivors may experience long-term sequelae, including chronic fatigue, arthralgia, and ocular complications such as uveitis, potentially linked to viral persistence [23,24].

### 3.1. Present and Future in the Therapy of EVD

During the 2018–2020 outbreak of EBOV in the DRC, researchers launched a landmark clinical study—the first randomized, multi-drug trial for EVD. In November 2018, the World Health Organization (WHO) and its partners launched the PALM trial (NCT03719586), led jointly by the U.S. National Institutes of Health (NIH) and the Congolese Institut National de Recherche Biomédicale (INRB). The study tested four potential Ebola treatments—mAb114, REGN-EB3, ZMapp and remdesivir—with the NIH funding most of the drug production and trial operations, while Gilead Sciences supplied remdesivir. Despite the difficulties of running a large clinical trial amid an active Ebola outbreak, the study was successfully completed. By August 2019, results showed that two mAb therapies, mAb114 (ansuvimab) and REGN-EB3, were significantly more effective at lowering Ebola-related deaths, reducing mortality from about 50% with ZMapp to roughly 33%. The resulting evidence paved the way for the U.S. Food and Drug Administration (FDA) to authorize the two mAb treatments in late 2020 for use against EBOV infections in both adult and pediatric patients [25,26].

**Figure 1:**
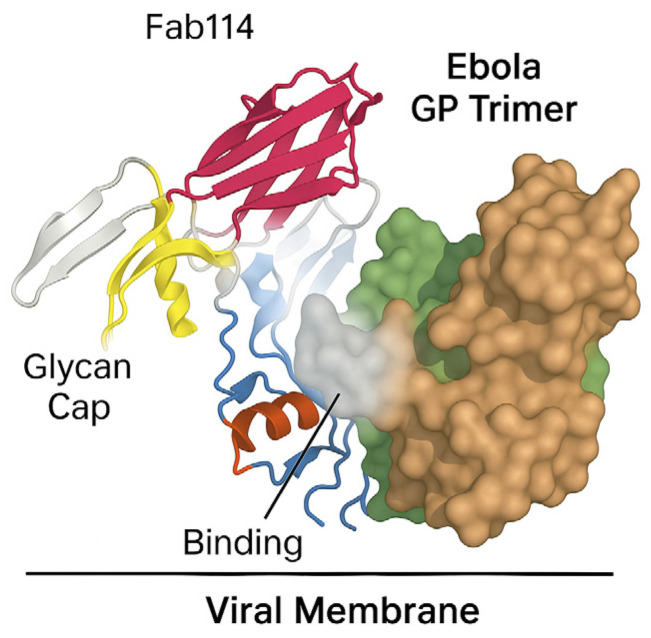
Fab114 interaction with the EBOV GP trimer.

Ansuvimab (mAb114) emerged from an international research partnership involving teams from NIH, INRB, the Institute for Research in Biomedicine in Switzerland, and the U.S. Army Medical Research Institute of Infectious Diseases. The discovery, co-led by scientists Nancy Sullivan and J. J. Muyembe-Tamfum, traces back to blood samples collected from a survivor of the 1995 Ebola outbreak in Kikwit, which served as the source of the antibody about ten years later [27]. Ansuvimab belongs to the IgG1 subclass of antibodies, featuring a variable heavy chain derived from the V3-1301 gene segment and a light chain from the VK1-2701 gene segment. Its heavy chain complementarity-determining region 3 is 13 amino acids long [28]. Regarding the mechanism of action, the fragment antigen-binding region (Fab region) derived from mAb114 recognizes a conserved amino acid region within the receptor-binding domain (RBD) of the EBOV glycoprotein (GP), targeting both the glycan cap and the core domain of the GP1 subunit (Figure 1) [29].

The Fab114 heavy chain is shown in magenta and the light chain in white, while the glycan cap is highlighted in yellow. The GP trimer is represented in brown and green, with one protomer shown as a ribbon to illustrate structural features of the glycoprotein. The binding interface between Fab114 and the Ebola GP is emphasized to highlight the region of molecular interaction. The illustration was created by the authors based on structural data reported by Tshiani Mbaya et al. [29]. This interaction site is directly relevant for the design and optimization of monoclonal antibody–based therapeutics, influencing antigen targeting, binding affinity, and formulation stability.

In late 2020, ansuvimab was approved by the FDA and it is currently marketed under the brand name Ebanga [30].

From a pharmaceutical perspective, ansuvimab presents several features that are highly relevant to its practical use in outbreak settings. It is supplied as a sterile, preservative-free lyophilized powder in single-dose vials, a format chosen to improve product stability during storage and transport. Before administration, the product must be reconstituted with sterile water and subsequently diluted in either 0.9% sodium chloride or 5% dextrose solution for intravenous infusion. The recommended dose is 50 mg/kg given as a single IV infusion over 60 min, which simplifies the dosing schedule but still requires infusion infrastructure and trained healthcare personnel. Preparation must be performed carefully under aseptic conditions, with gentle swirling rather than shaking, and both the reconstituted and diluted solutions have only limited refrigerated in-use stability, which adds logistical complexity during emergency deployment. In addition, the product must be stored at 2–8 °C, protected from light, and must not be frozen or shaken, underscoring its dependence on a reliable cold chain. Available pharmacokinetic data from healthy volunteers indicate a profile generally consistent with that of other IgG1 monoclonal antibodies, although data in Ebola-infected patients remain limited. Overall, ansuvimab illustrates how the clinical utility of an effective antiviral biologic is closely linked not only to its antiviral activity, but also to formulation design, storage conditions, preparation steps, and infusion-related delivery requirements [31].

**Figure 2:**
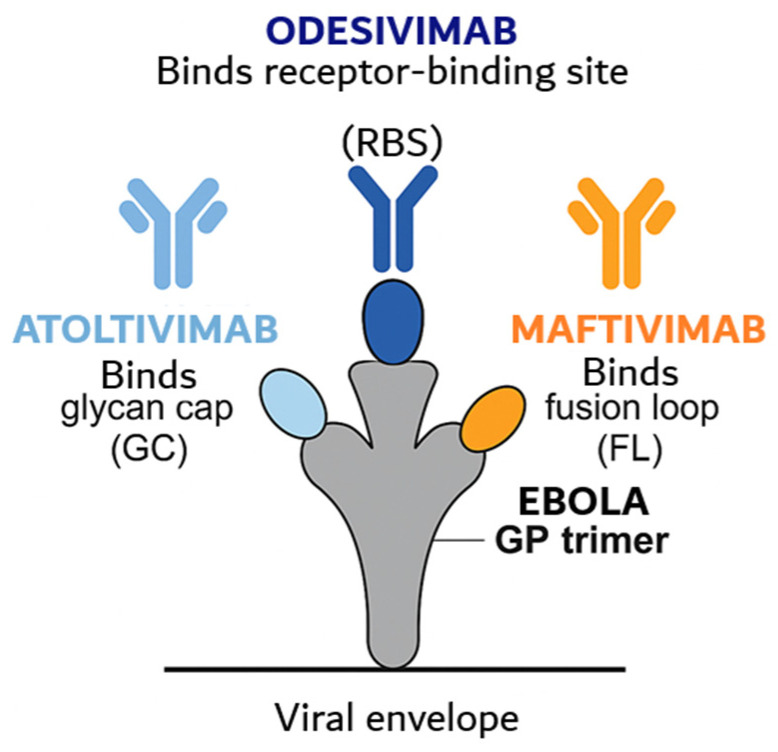
Schematic representation of the interaction between EBOV GP and the monoclonal antibodies of the REGN-EB3 therapeutic cocktail (atoltivimab, odesivimab, and maftivimab). These antibodies bind distinct epitopes on the glycoprotein, including the glycan cap (GC), receptor-binding site (RBS), and fusion loop (FL). The illustration was created by the authors based on structural and mechanistic data reported by Rayaprolu et al. [32]. Targeting multiple non-overlapping epitopes is relevant for therapeutic efficacy and resistance prevention, and has important implications for antibody formulation, dosing strategies, and combination design.

REGN-EB3 consists of three fully human monoclonal antibodies—atoltivimab (REGN3470), odesivimab (REGN3471), and maftivimab (REGN3479)—that each recognize separate, non-competing sites on the Ebola GP (Figure 2) [32].

Acting together, they prevent the virus from attaching to and entering host cells. In vitro, atoltivimab and odesivimab exhibit weak neutralizing activity but contribute through Fc-mediated functions, whereas maftivimab displays strong neutralizing potency [33,34,35]. Treatment with REGN-EB3 was associated with a 17% reduction in EBOLA mortality. The most reported infusion-related adverse effects (at least 20% of subjects who received the treatment) were fever, chills, tachycardia, tachypnea, and vomiting. Other adverse effects like diarrhea or hypoxia were much rarer [36,37].

In October 2020, REGN-EB3 was approved by the FDA and it is currently marketed by Regeneron under the brand name Inmazeb [37]. The product is supplied as a sterile, preservative-free injectable solution and is available in two concentration presentations containing equal amounts of the three monoclonal antibodies. The formulation includes stabilizing excipients commonly used in antibody preparations, such as histidine buffer components, sucrose, and polysorbate-80, which help maintain protein stability during storage and handling. The recommended therapeutic regimen consists of 50 mg/kg of each antibody administered as a single intravenous infusion after dilution in compatible infusion solutions, including sodium chloride, dextrose, or lactated Ringer’s solution. Preparation must be performed under medical supervision and involves dilution in an intravenous infusion bag followed by administration through an infusion line equipped with a 0.2 μm filter. Similar to other monoclonal antibody therapeutics, the product requires refrigerated storage at 2–8 °C and protection from light, and the diluted infusion solution has limited stability, which necessitates relatively prompt administration after preparation. Pharmacokinetic studies in healthy volunteers indicate dose-proportional systemic exposure and elimination half-lives of approximately 21–25 days, consistent with the prolonged circulation profiles characteristic of IgG antibodies. As with other biologic countermeasures for Ebola virus disease, the need for infusion-based administration and cold-chain maintenance represents an important logistical consideration during outbreak deployment [38].

Ansuvimab and REGN-EB3 are currently the only therapeutics approved for the treatment of EVD. However, additional candidate molecules are under investigation in various stages of clinical and preclinical development. A summary of the investigational agents tested so far is presented in Table 1.

#### Comparative Perspective on Investigational Antiviral Strategies

The investigational antiviral agents summarized in Table 1 reflect a diverse range of therapeutic strategies targeting different stages of the Ebola virus life cycle. From a pharmaceutics perspective, these approaches differ substantially in terms of delivery requirements, pharmacokinetic behavior, manufacturability, and feasibility of deployment in outbreak settings.

Broad-spectrum nucleoside analogues such as remdesivir, favipiravir, galidesivir, and obeldesivir primarily inhibit the viral RNA-dependent RNA polymerase, thereby suppressing viral replication. These small-molecule antivirals offer advantages related to formulation flexibility and, in some cases, the potential for non-intravenous or oral administration. However, their clinical performance has been inconsistent, partly due to pharmacokinetic limitations, including suboptimal systemic exposure and challenges in maintaining therapeutic concentrations in advanced disease stages. While their relatively simpler manufacturing processes and scalability are advantageous, these benefits have not consistently translated into robust clinical efficacy [54,55].

In contrast, monoclonal antibody-based therapies such as ZMapp and MBP134AF provide highly specific viral neutralization and have demonstrated superior efficacy in both preclinical and clinical settings. From a pharmacokinetic perspective, these biologics offer prolonged systemic exposure and high target specificity; however, their use is constrained by intravenous administration, cold-chain dependence, and limited stability following reconstitution. In addition, large-scale production requires complex biomanufacturing infrastructure, which may limit rapid deployment during outbreaks, particularly in resource-limited settings [55,56].

RNA-targeting strategies, including siRNA-based therapeutics such as TKM-130803, represent a mechanistically distinct approach by directly interfering with viral gene expression. Despite their high specificity, their clinical translation remains limited by delivery challenges, including the need for nanoparticle-based carriers, stability concerns, and variable in vivo pharmacodynamics [53].

Overall, the comparative evaluation of these antiviral classes highlights a fundamental trade-off between antiviral potency and feasibility of use. Monoclonal antibodies currently provide the most consistent efficacy but are limited by delivery and manufacturing constraints, whereas small-molecule antivirals and nucleic acid-based approaches offer advantages in manufacturing scalability and potential ease of administration but face pharmacokinetic and efficacy-related limitations. From a pharmaceutics standpoint, future therapeutic development should prioritize strategies that integrate potent antiviral activity with optimized pharmacokinetic profiles, simplified delivery systems, and improved stability to enable effective use in outbreak settings.

### 3.2. Approved and Experimental Vaccines for EVD

Two vaccines have been approved for Ebola prevention and prequalified by the WHO: the single-dose Ervebo and the two-dose Zabdeno/Mvabea regimen. Other two available vaccines are approved in China and Russia.

Ervebo (rVSV-EBOV) is a live, attenuated, replication-competent vaccine engineered from vesicular stomatitis virus (VSV) to express the glycoprotein of the EBOV. Administered as a single dose, it demonstrated strong safety, immunogenicity, and long-lasting antibody responses—persisting for up to two years—in phase 2 and 3 clinical trials. Drawing on the successful outcomes observed during the 2014–2016 Ebola outbreak in Guinea and its implementation during the tenth outbreak in the eastern Kivu region of the DRC—where over 28,000 individuals were vaccinated to curb ongoing human transmission—Ervebo was subsequently granted FDA and the European Medicines Agency (EMA) approval in 2019 for the prevention of Ebola virus disease in adults, and FDA approval in 2023 for administration to children aged one year and older [51,57,58].

Zabdeno/Mvabea (Ad26.ZEBOV/MVA-BN-Filo vaccine regimen) is a two-dose heterologous Ebola vaccine developed by Janssen Vaccines & Prevention B.V. in collaboration with Bavarian Nordic. The first dose administered is Ad26.ZEBOV (Zabdeno), a monovalent, replication-incompetent adenoviral vector serotype 26 (Ad26) vaccine encoding the full-length GP of the EBOV Mayinga variant. The second dose, administered approximately eight weeks later, is MVA-BN-Filo (Mvabea), a nonreplicating, multivalent modified vaccinia Ankara (MVA) vaccine that encodes the EBOV Mayinga GP, the Taï Forest ebolavirus nucleoprotein, the Sudan ebolavirus Gulu GP, and the Marburg virus Musoke GP [59].

Clinical trials have shown the Zabdeno/Mvabea regimen to be safe and immunogenic, eliciting robust and durable antibody and T-cell responses [60]. Although classical efficacy studies have not been feasible, immunobridging analyses from nonhuman primate data to human responses support a high likelihood of protective efficacy, leading to marketing authorization by EMA in May 2020, under exceptional circumstances, for prophylactic vaccination in children and adults [59,61].

However, this regimen is less suitable for outbreak response, as two doses administered eight weeks apart are required before full protection develops [62]. Additionally, preexisting immunity to the Ad26 vector may theoretically reduce vaccine effectiveness. Consequently, while Zabdeno/Mvabea offers a valuable tool for preventive immunization, its efficacy is lower than that of Ervebo, and its use remains limited to special conditions [51].

The Ad5-EBOV vaccine is a recombinant adenovirus type 5 (Ad5) vector-based Ebola vaccine co-developed by the Bioengineering Institute of the Chinese Academy of Military Medical Sciences in collaboration with Tianjin CanSino Biotechnology Inc. In 2017, it received licensure from the China Food and Drug Administration (CFDA), for emergency use [63].

Administered as a single-dose vaccine, Ad5-EBOV has demonstrated good safety and immunogenicity profiles in clinical studies, inducing specific glycoprotein (GP)-targeted antibody and T-cell responses within 14 days of vaccination. Reported adverse events were mild and self-limiting [64].

However, a major limitation of this vaccine lies in its use of the Ad5 viral vector, as preexisting immunity to adenovirus type 5 is common in the general population. This preexisting seropositivity may diminish the vaccine’s immunogenicity and protective efficacy by interfering with the vector’s ability to deliver the Ebola GP antigen effectively [65].

The GamEvac-Combi vaccine is a bivalent EVD vaccine that employs recombinant VSV and Ad5 vectors designed to induce protective immune responses. In preclinical evaluations, the vaccine demonstrated complete protection in non-human primates against a lethal EBOV challenge, both four weeks after vaccination and five months after initiation of the immunization course. Subsequent phase I/II clinical trials in Russia confirmed the safety and strong immunogenicity of GamEvac-Combi in humans, which ultimately led to its regulatory approval in the Russian Federation [66].

Further validation came from a recent randomized, double-blind, placebo-controlled clinical study carried out in both the Republic of Guinea and Russia. Results revealed that vaccinated individuals mounted robust GP-specific interferon-γ (IFN-γ) responses by day 28, along with neutralizing antibody activity detected by day 42. Moreover, GP-specific IgG antibody concentrations peaked at day 42 and remained elevated for up to one year, indicating that the vaccine is capable of inducing sustained humoral and cellular immunity [67].

In addition to the approved vaccines, several experimental candidates are under investigation. The most significant ones are presented in Table 2.

#### Comparative Perspective on Emerging Ebola Vaccine Platforms

The vaccine platforms currently under investigation for Ebola virus disease encompass a range of technological approaches, including viral vector-based vaccines, recombinant protein formulations, and nucleic acid-based strategies (see Table 2). From a pharmaceutics perspective, these platforms differ substantially in terms of immunogenicity, stability, manufacturing complexity, and deployment feasibility.

Viral vector-based vaccines, such as recombinant vesicular stomatitis virus (rVSV)–based candidates, have demonstrated robust and rapid immune responses, often after a single dose, making them particularly suitable for outbreak control. However, their use is associated with specific pharmaceutical and logistical constraints, including cold-chain requirements, limited thermostability, and challenges related to large-scale production and distribution under emergency conditions. In addition, vector-related safety considerations and pre-existing immunity may influence their effectiveness in certain populations [79].

Recombinant protein-based vaccines, including those formulated with adjuvants such as Matrix-M, offer advantages in terms of safety profile and formulation flexibility. These platforms are generally more amenable to stabilization strategies and may exhibit improved storage characteristics compared to live viral vectors. However, they typically require multiple doses and adjuvant systems to achieve adequate immunogenicity, which can complicate deployment during outbreaks and increase logistical burden [80].

Nucleic acid-based vaccine platforms, including DNA and mRNA vaccines, represent a highly adaptable approach with significant potential for rapid design and scalable manufacturing. From a formulation perspective, these technologies benefit from platform-based production and the possibility of standardized manufacturing processes. However, their clinical performance and durability of immune responses in the context of filovirus infections remain under active investigation. In addition, delivery systems—such as lipid nanoparticles for mRNA vaccines—introduce stability and storage challenges that may limit deployment in resource-constrained environments [78].

Overall, the comparative evaluation of Ebola vaccine platforms reveals important trade-offs between immunogenicity, safety, and feasibility of use. Viral vector-based vaccines currently offer the most rapid and effective immune protection but are constrained by storage and distribution requirements. In contrast, protein-based and nucleic acid-based platforms provide advantages in manufacturing scalability and formulation flexibility but may require optimization to achieve comparable efficacy and field applicability. From a pharmaceutics standpoint, future vaccine development should focus on improving thermostability, simplifying dosing regimens, and ensuring scalable production to enable rapid and effective deployment during outbreaks.

## 4. Sudan Virus Disease (SVD)

Sudan virus disease (SVD), caused by the Sudan virus (SUDV) (*Orthoebolavirus sudanense*), shares its presumed reservoir host and clinical manifestations with EVD. However, SVD is characterized by comparatively lower transmissibility and a more limited geographic distribution, primarily confined to Sudan and Uganda [81,82].

SVD was first identified in 1976 in South Sudan, resulting in 284 cases and 151 deaths. Since then, seven additional outbreaks (1979, 2000, 2004, 2011, 2012, 2022, and 2025) have been reported, totaling 659 cases and 316 deaths. The largest outbreak occurred in Uganda in 2000, with 425 confirmed cases and 224 fatalities. The most recent outbreak, also in Uganda, involved 12 cases and 4 deaths and was declared over on 26 April 2025 [10,83].

### 4.1. Therapeutic Approaches and Future Directions for SVD

Currently, no targeted therapeutic options are available for SVD, but several investigations have provided evidence that certain candidate drugs exhibit efficacy against SUDV.

Findings from the PALM trial indicated that, although remdesivir yielded a modest survival rate of approximately 47% in EVD, its performance suggested that pairing it with antibody-based therapeutics may offer enhanced clinical benefit (see Table 1).

Given its global availability and the extensive safety data accumulated during its use for COVID-19, the WHO recommended including remdesivir in future randomized controlled trials to assess its therapeutic value against SVD, both as a standalone agent and in combination with monoclonal antibodies [81].

**Figure 3:**
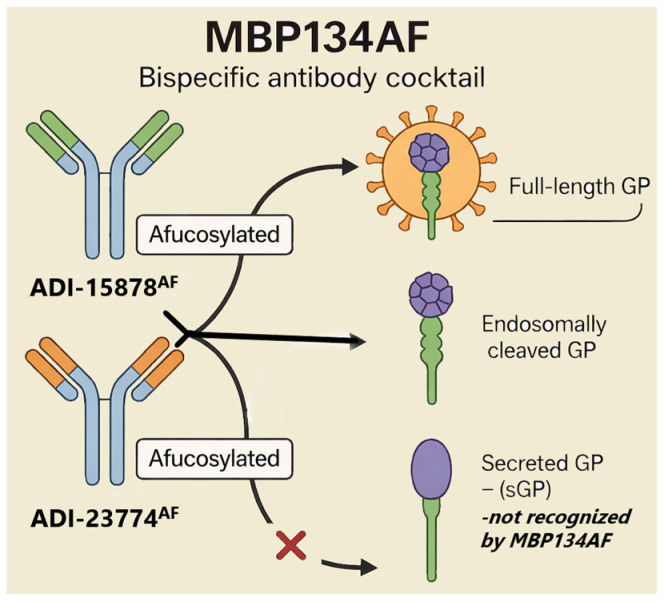
Schematic overview of the MBP134AF antibody cocktail illustrating the broadly neutralizing antibodies ADI-15878AF and ADI-23774AF and their recognition of distinct ebolavirus glycoprotein (GP) forms, including the full-length virion-associated GP and the endosomally cleaved GP involved in viral entry, while the secreted GP isoform (sGP) is not recognized. Illustration created by the authors based on information reported in the literature [84,85]. Selective targeting of functional GP forms while avoiding sGP is relevant for optimizing therapeutic specificity and may have important implications for antibody efficacy, dosing strategies, and formulation development.

MBP134AF is a bispecific antibody cocktail composed of the broadly neutralizing antibodies ADI-15878AF and ADI-23774AF. As described above for Ebola-directed antibody therapies, these antibodies recognize distinct, non-overlapping epitopes on ebolavirus glycoprotein and selectively target membrane-associated GP forms rather than the secreted GP isoform (sGP) (Figure 3), a feature expected to enhance therapeutic specificity and pharmacological performance [84,85].

Researchers at Mapp Biopharmaceutical reported that a single 7.5 mg/kg administration of MBP134 provided complete protection in non-human primates (NHPs) exposed to the SUDV Boniface variant. In a subsequent fully blinded efficacy study using a rhesus macaque model challenged with the highly lethal SUDV Gulu variant, MBP134 was able to control established disease following a single dose administered either intravenously or intramuscularly, even when treatment was initiated up to five days after viral challenge. These findings highlight the promising therapeutic activity of MBP134 in preclinical models, although further clinical evaluation is required to determine its effectiveness in humans [42,84].

Based on these results and outcomes observed early in the 2022 Uganda outbreak, during which a small number of patients were successfully treated with MBP134 and remdesivir [86], the Uganda Ministry of Health authorized the expanded-access use of these two experimental drugs at the end of 2022 for eligible patients who provided informed consent [87].

Another therapeutic strategy for SUDV involves a combination of two chimeric monoclonal antibodies generated in NHPs and engineered with human Fc domains. Rather than repeating the general principles of antibody-mediated neutralization, it is sufficient to note here that FVM04 binds the receptor-binding site of ebolavirus GP and blocks its interaction with the endosomal entry receptor Niemann–Pick C1 (NPC1), whereas *CA45* targets a conserved internal fusion loop shared across ebolaviruses [88,89].

Recent work demonstrated robust therapeutic efficacy of the FVM04/CA45 antibody cocktail in cynomolgus macaques challenged with SUDV, even when treatment was delayed. Administration beginning five days after infection, followed by a second dose on day eight, resulted in minimal clinical disease and complete survival of treated animals. These findings highlight the strong potential of the FVM04/CA45 cocktail as a delayed-intervention therapy for SUDV infection [90].

Obeldesivir (see Table 1) has also been evaluated as a therapeutic candidate for Sudan virus disease. Once-daily oral administration of obeldesivir to cynomolgus macaques, initiated 24 h after SUDV challenge and continued for 10 days, resulted in complete protection from lethal infection. In these studies, a full 10-day regimen provided 100% survival, whereas a shortened 5-day course still conferred substantial benefit, with 60% of animals surviving an otherwise uniformly fatal disease. Notably, viral rebound was observed soon after treatment discontinuation in the 5-day cohort, while the 10-day regimen was sufficient to suppress viremia until a protective adaptive immune response was established [91].

### 4.2. Vaccine Approaches and Future Directions for SVD

No vaccines are currently available for use against SVD; several SUDV vaccine candidates are in development, but none have yet received regulatory approval.

The rapid progression of the 2022 SUDV outbreak in Uganda prompted the WHO and the Ugandan Ministry of Health to jointly initiate a randomized ring-vaccination trial to assess one or more investigational vaccine candidates. In November 2022, the WHO Vaccine Prioritization Working Group identified three SUDV vaccine candidates—rVSV-SUDV, ChAd3-SUDV, and ChAdOx1-biEBOV—for use in the planned vaccination campaign in Uganda [92].

Among these, *rVSV-SUDV* received the highest priority. Originally developed by Merck and subsequently advanced by the International AIDS Vaccine Initiative (IAVI), it employs the same recombinant vesicular stomatitis virus (rVSV) vector platform used for Ervebo. As discussed for Ebola vaccines (Section 3.2), this platform is characterized by rapid immunogenicity and suitability for outbreak response. The rVSV-SUDV candidate is a replication-competent, live-attenuated construct in which the VSV G (ΔG) gene is replaced with the gene encoding the SUDV GP. Preclinical challenge studies demonstrated complete protection of vaccinated animals [81]. A Phase 1, single-blind, placebo-controlled, dose-escalation trial conducted between 2023 and 2024 found that a single dose of rVSVΔG-SEBOV-GP was safe, well tolerated, and generated immune responses persisting for at least six months [93].

In 2025, WHO placed priority on evaluating IAVI’s SUDV vaccine, which had been pre-positioned in Uganda as part of the international response to the country’s SVD outbreak. The resulting ring-vaccination study—TOKEMEZA SVD—was launched on 3 February 2025, only four days after the outbreak declaration [94]. Following confirmation of the first case on 30 January, investigators from Makerere University and the Uganda Virus Research Institute, supported by the WHO, implemented the trial. Contacts of each confirmed case were organized into rings that were subsequently randomized to immediate or delayed vaccination, consistent with the established ring-vaccination design used to evaluate vaccine efficacy, safety, and immunogenicity [95]. A minimum of 17 rings were defined and randomized during the TOKEMEZA SVD trial. Data generated from this study will guide forthcoming stages of IAVI’s SUDV vaccine research and development program [94].

Another VSV-based vaccine candidate targeting SUDV, engineered to express the glycoprotein of the SUDV-Gulu strain, has recently advanced through preclinical assessment. Vaccinated non-human primates (NHPs) generated robust GP-specific IgG responses within two weeks, including measurable cross-reactivity. Upon subsequent challenge with a lethal dose of SUDV, animals receiving the VSV-SUDV vaccine showed complete protection and no evidence of clinical disease in this experimental model [96,97].

ChAd3-SUDV is based on a non-replicating chimpanzee adenovirus serotype 3 vector and was developed through efforts led by the Sabin Vaccine Institute together with the U.S. National Institutes of Health. As with other adenoviral vector vaccines described in Section 3.2, preclinical evaluation in NHPs indicated that a single immunization was associated with both rapid and sustained protection against otherwise lethal SUDV exposure, with protection closely linked to the magnitude of GP-specific antibody responses [98,99]. These findings support the further development of ChAd3-SUDV as a vaccine candidate, although additional clinical studies are required to determine its protective efficacy in humans. Early clinical investigations (Phase I/Ib) further support its potential, indicating that the vaccine is well tolerated and elicits a vigorous immune response. Antibody titers rise rapidly, reaching their peak within 2–4 weeks after vaccination, with seroconversion achieved in all participants during this period. Notably, these antibody responses remain measurable for at least one year following immunization [100].

ChAdOx1 biEBOV developed by Jenner Institute and Oxford Vaccine Group, is a bivalent filovirus vaccine based on the replication-deficient simian adenoviral vector ChAdOx1. This platform has been widely evaluated in other vaccine contexts, as outlined in Section 3.2. The construct co-expresses the glycoproteins of SUDV and EBOV, achieved by insertion of the SUDV GP gene at the E1 locus and the EBOV GP gene at the E4 locus [101]. Clinical assessment has included a Phase 1 trial in the United Kingdom (2021) and a Phase 1b study in Tanzania (2022), enabling evaluation of the vaccine in both non-endemic and endemic-region populations. Across these studies, ChAdOx1 biEBOV demonstrated a favorable safety profile and was generally well tolerated, consistent with other vaccines using the same adenoviral backbone. A single dose elicited binding antibodies against both SUDV and EBOV GPs, and booster administration further augmented these responses, supporting continued development of this bivalent vaccine candidate [101,102].

A promising SUDV vaccine candidate has recently been developed using a nanocarrier platform (LION™) formulated with an alphavirus-derived replicon RNA. As with other nucleic acid–based approaches described earlier, a single dose of this LION-SUDV formulation conferred uniform protection in guinea pigs challenged with a lethal SUDV exposure. Vaccinated animals exhibited no clinical signs of disease, undetectable viremia, and an absence of measurable viral burden in tissues, alongside a broad, antigen-specific humoral response. These findings support further evaluation of this vaccine in additional animal models to more fully define its preclinical efficacy [103].

## 5. Bundibugyo Virus Disease (BVD)

Bundibugyo virus disease (BVD) is caused by Bundibugyo ebolavirus (BDBV; *Orthoebolavirus bundibugyoense*), a member of the *Filoviridae* family (Table S1). To date, only two BVD outbreaks have been documented. The first occurred in 2007 in the Bundibugyo district of Uganda and involved 131 reported cases with 42 deaths. A second outbreak was identified in 2012 in the DRC, resulting in 38 cases and 13 fatalities. The corresponding case-fatality rates—approximately 42% and 34%—are notably lower than those typically associated with EBOV and SUDV epidemics [10,104]. Despite the significant clinical impact of BDBV infection, the virus has received relatively little emphasis in global health priorities. As a result, survivors of BDBV disease have been largely left out of the research initiatives and intervention programs that have advanced treatments, vaccine development, and clinical care for other Ebola virus species [105]. However, promising advances have been made toward the development of effective vaccines against BDBV.

In a notable study, a recombinant VSV-based candidate, rVSVΔG/BDBV-GP, was evaluated in cynomolgus macaques challenged with a lethal dose of BDBV. The vaccine was administered approximately 20 min post-infection. This intervention resulted in 83% survival, markedly higher than the ~21% natural survival rate typically observed in this macaque model. Animals receiving the vaccine displayed a rapid onset of circulating immune activity, in contrast to the untreated control, which showed no measurable response. Among the vaccinated cohort, those that survived generated detectable GP-targeted IgM followed by IgG, whereas the animals that did not survive showed little to no IgG production. Collectively, these findings reinforce the potential of rVSV-based platforms as effective post-exposure prophylactic countermeasures against filoviruses. Notably, the results indicate that this approach is also effective against BDBV when administered promptly after exposure [106].

A distinct vaccination strategy against BDBV has utilized a human parainfluenza virus type 3 (HPIV3) vector expressing the BDBV Uganda isolate GP (KU182911.1). Intranasal immunization of ferrets with this monovalent HPIV3-BDBV construct resulted in complete protection. All vaccinated animals survived the challenge and exhibited neither weight loss nor clinical signs of disease throughout the monitoring period. Moreover, no vaccinated ferrets developed detectable viremia, indicating robust control of viral replication [107].

## 6. Taï Forest Virus Disease (TAFD)

Taï Forest virus (TAFV), a comparatively understudied member of the *Filoviridae*, was first detected in 1994 during multiple chimpanzee outbreaks in Côte d’Ivoire’s Parc National de Taï [108]. Human infection has been documented only once, in a case linked to contact with an infected chimpanzee, confirming the virus’s capacity to cause hemorrhagic disease in humans. Despite its pathogenic potential, research on TAFV remains limited, and no standardized small-animal models have yet been established for experimental studies [109]. In efforts to develop an effective vaccine against this infection, VSV-based platforms currently represent the most promising strategy and, at present, remain the sole established approach under active investigation [110].

A recent investigation evaluated a TAFV vaccine candidate built on the VSV vector platform and incorporating the TAFV glycoprotein as the immunogen. *Cynomolgus macaques* received a single high dose of the VSV-TAFV construct prior to lethal viral challenge. In this experimental model, the single-dose regimen resulted in complete protection in non-human primates, suggesting that VSV-based platforms may represent a promising strategy for TAFV vaccine development, although further evaluation in clinical settings is required [108].

## 7. Marburg Virus Disease (MVD)

**Figure 4:**
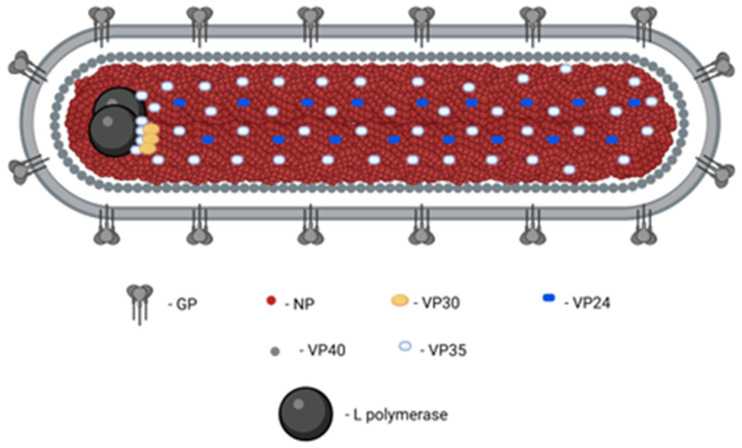
Schematic representation of the Marburg virus (MARV) virion showing the major structural proteins associated with the viral envelope and nucleocapsid, including GP, NP, VP24, VP30, VP35, VP40, and the L polymerase. Created with BioRender.com. These structural components define key targets for antiviral drug development and vaccine design and are relevant for the identification of antigenic sites and formulation strategies.

MVD is a severe and often lethal disorder exhibiting a wide and dynamic range of clinical manifestations. It is caused by another member of *Filoviridae* family, Marburg virus (MARV), which was the first filovirus discovered and was identified by electron microscopy in 1967 [111]. MARV is a negative-sense, single-stranded RNA virus (~19 kb) encoding structural proteins involved in viral replication, immune evasion, and host-cell entry, including glycoprotein-mediated attachment and fusion (Figure 4) [112].

MVD occurs primarily in Sub-Saharan Africa (Table S1), where most outbreaks have been reported. According to CDC data, 19 documented outbreaks have resulted in 679 cases and 422 deaths, with the largest outbreak occurring in Angola (2004–2005). More recent cases, including those reported in Tanzania in 2025, highlight the continued outbreak potential of MARV [113].

Transmission is typically zoonotic, associated with exposure to the natural reservoir, the fruit bat *Rousettus aegyptiacus*, with subsequent human-to-human transmission occurring via contact with infected bodily fluids or contaminated materials. Nosocomial spread represents a significant risk in settings with inadequate infection control [111,114,115].

Following an incubation period of 2–21 days, MARV infects immune cells such as macrophages and dendritic cells, with subsequent systemic dissemination to multiple organs. Clinically, MVD presents with acute febrile illness, including headache, myalgia, and gastrointestinal symptoms, which may progress to severe hemorrhagic manifestations, neurological involvement, and multi-organ failure [116,117,118].

### 7.1. Therapeutic Approaches and Future Directions for MVD

Current investigational approaches for the management of MVD predominantly center on antiviral agents—many of which have demonstrated therapeutic potential against other *Filoviridae* infections—as well as on the development of mAb-based interventions.

Thus, remdesivir (see Table 1) demonstrated therapeutic benefit in cynomolgus macaques experimentally infected with MARV. Treatment initiated several days after infection markedly improved survival, and treated animals exhibited better clinical outcomes, including reduced plasma viral RNA levels and improved indicators of renal function, hepatic function, and coagulation, relative to untreated animals [119]. Other preclinical investigations have shown that combining remdesivir with selected monoclonal antibodies confers enhanced protection in MARV-infected rhesus macaques, yielding superior outcomes relative to remdesivir monotherapy [120]. In late September 2024, Rwanda documented an outbreak of MVD. Intravenous remdesivir was provided to 52 affected individuals, including all patients requiring advanced respiratory or organ support. Mortality among those receiving remdesivir was 6%, whereas deaths occurred in the majority of untreated patients. However, the absence of randomized treatment allocation limits interpretation, as additional clinical or epidemiologic factors may have influenced the observed survival advantage. In the same outbreak, remdesivir was also administered as post-exposure prophylaxis to more than 150 healthcare personnel [121]. During the 2023 MVD outbreak in Equatorial Guinea, four patients received remdesivir, with only one fatal outcome reported [122].

Several antivirals previously investigated for activity against other filoviruses, including EBOV and SUDV, have likewise demonstrated potential efficacy against MARV.

In vitro experiments showed that favipiravir (see Table 1) inhibited MARV replication in Vero E6 cells, and subsequent studies in murine and nonhuman primate models confirmed its therapeutic activity. Notably, in mice, favipiravir afforded complete protection when treatment was initiated early after infection [123], and intravenous administration provided a significant survival advantage in cynomolgus macaques [124].

The antiviral potential of galidesivir (see Table 1) has been demonstrated in animal models of MARV infection. In guinea pigs, intraperitoneal administration initiated within 48 h of exposure conferred significant protection [47]. Further evaluation in cynomolgus macaques showed that higher-dose galidesivir, delivered twice daily beginning 24 or 48 h after challenge, markedly suppressed viremia and mitigated clinical manifestations. This therapeutic approach resulted in complete survival of all treated animals [125,126].

A recent investigation demonstrated that oral obeldesivir (see Table 1) confers substantial protection in preclinical models of MVD. In cynomolgus macaques, once-daily treatment begun 24 h after viral exposure and continued for 10 days achieved 80% survival following a highly lethal MARV challenge, while also delaying viral replication and disease progression [127].

MBP091 is an investigational monoclonal antibody targeting MARV glycoprotein. As discussed for other filovirus-directed antibody therapies (Section 3.1), such antibodies neutralize viral entry by binding critical regions of the glycoprotein. In this context, MBP091 binds a conserved receptor-binding site and has demonstrated efficacy across multiple animal models. In guinea pigs infected with an adapted MARV strain and in rhesus macaques challenged with MARV/Angola, MBP091 conferred robust protection, highlighting its therapeutic potential [125,128]. During the 2024 MVD outbreak in Rwanda, MBP091 was administered to a cohort of 10 patients. Of these, six individuals received the antibody through an expanded-access protocol, whereas four were treated after enrollment in a WHO-coordinated randomized clinical study [129]. Overall, 2 of the 10 MBP091-treated patients (10%) succumbed to the illness, in contrast with 13 of 56 patients (23%) who did not receive the investigational therapy [121].

MR186-YTE is an engineered monoclonal antibody designed to enhance Fc-mediated effector functions. Rather than repeating general antibody engineering principles, it is noted here that administration of MR186-YTE at 5 days post-inoculation afforded complete protection from lethal challenge, whereas administration at 6 days post-inoculation did not confer survival benefit. In contrast, the combination of MR186-YTE with remdesivir markedly improved outcomes at this later intervention point, with four of five animals surviving and exhibiting rapid clinical recovery alongside a pronounced reduction in peripheral viremia [120].

siRNA-based approaches represent an alternative antiviral strategy targeting viral gene expression, as outlined in Section 3.1. In this context, an investigational therapeutic comprising a sequence-specific siRNA (NP-718 m) encapsulated within lipid nanoparticles (LNPs) demonstrated broad protection against multiple MARV strains in guinea pig models [130], and in subsequent evaluations in rhesus macaques resulted in markedly attenuated clinical disease and significantly reduced viremia relative to untreated controls [131].

More recently, second-generation siRNA platforms employing ligand conjugation—specifically mannose and N-acetylgalactosamine (GalNAc)—have been developed. These conjugates are compatible with subcutaneous administration and exhibit greater chemical and biological stability compared with LNP-based formulations. Although each conjugate provided only modest survival benefit when administered individually in MARV-Angola–infected guinea pigs, combined administration achieved up to 100% survival when delivered 24 h post-infection [132].

Additional therapeutic strategies for MVD remain under active investigation:

*AVI-7288*, a positively charged phosphorodiamidate morpholino oligomer directed against MARV nucleoprotein mRNA, has demonstrated post-exposure efficacy in NHP models, supported by favorable pharmacokinetic data in humans. These findings suggest that AVI-7288 may represent a promising candidate for prophylactic intervention against MARV infection, although further clinical evaluation is required to determine its effectiveness in humans [133,134].

**Figure 5:**
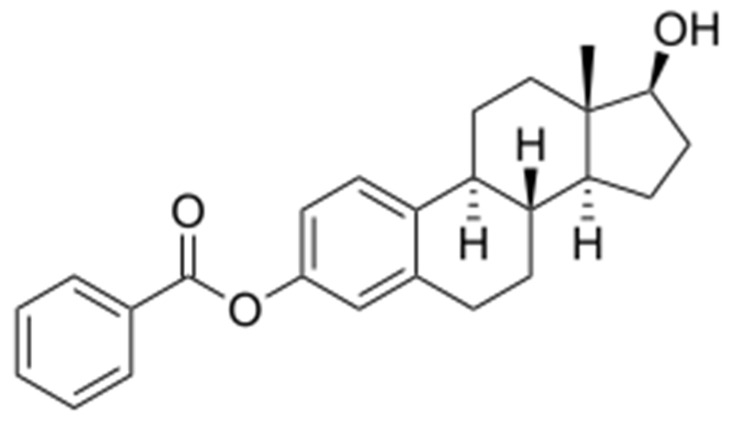
Chemical structure of estradiol benzoate.

Estradiol benzoate has also emerged as a candidate antiviral agent (Figure 5).

Computational and molecular studies show that it forms stable covalent interactions with the viral VP35 protein and exhibits strong binding affinity and favorable binding energetics. These findings suggest potential inhibition of VP35-mediated functions, including viral replication and immune evasion, positioning estradiol benzoate as a promising therapeutic lead for MARV [135].

**Figure 6:**
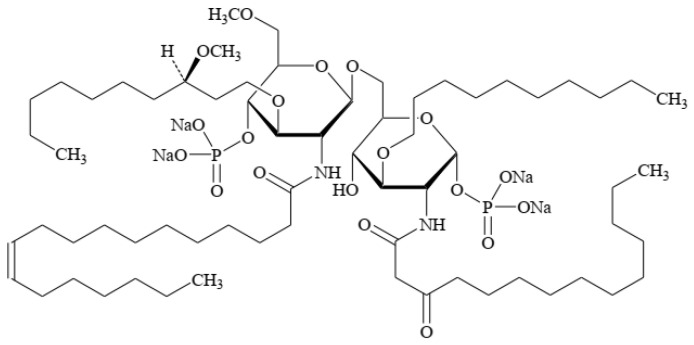
Chemical structure of Eritoran tetrasodium.

Eritoran tetrasodium (E5564) (Figure 6) offers another mechanistic approach.

As a Toll-like receptor 4 (TLR4) antagonist, eritoran mitigates dysregulated host inflammatory responses associated with severe MVD. In a MARV challenge study, mice receiving a 10-day post-infection course achieved 90% survival, compared with 20% in placebo-treated controls [136].

### 7.2. Investigational Vaccine Candidates for MARV

To date, no MARV vaccine has received regulatory authorization for human use. Nevertheless, several investigational candidates have demonstrated encouraging protective efficacy in non-human primate models, including cross-protection against both MARV and RAVN [125]. Based on predefined assessment criteria, the WHO Technical Advisory Group on vaccine candidate prioritization (October 2024) identified four vaccines for progression into subsequent developmental stages. These include a ChAd3-vectored platform from the Sabin Vaccine Institute, two VSV-vectored candidates—one developed by Public Health Vaccines (USA) and the other by the International AIDS Vaccine Initiative, along with a ChAdOx1-vectored vaccine produced by the University of Oxford [115,137].

The ChAd3 platform is a replication-deficient chimpanzee adenoviral vector engineered to express MARV surface GP. As described for other adenoviral vector vaccines (Section 3.2), this platform enables induction of antigen-specific immune responses without productive infection of host cells [138]. The vaccine was originally developed by the NIH Vaccine Research Center in partnership with GlaxoSmithKline Biologicals to support eventual U.S. FDA licensure, and its continued advancement is now led by the Sabin Vaccine Institute [139]. ChAd3 has undergone several preclinical evaluations involving 157 vaccinated NHPs. These studies indicated rapid and sustained protective effects in this experimental model, with partial protection observed as early as three days post-vaccination (60%) and complete protection by day seven. Long-term protective effects were also observed, with 100% protection at six months and 75% at twelve months. GP-binding antibody levels were identified as a potential correlate of protection. According to the Sabin Vaccine Institute, Phase 1 clinical studies have been completed, confirming the vaccine’s safety and immunogenicity in humans. Phase 2 evaluations have been conducted in Uganda, Kenya, Rwanda, and the United States [140]. During the 2024 Rwanda outbreak, an open-label Phase 2 study of the Sabin ChAd3 vaccine was initiated, in which more than 1700 frontline and healthcare workers, as well as high-risk contacts, received the investigational vaccine and were closely monitored. The resulting data are contributing important evidence to support further development of Sabin’s vaccine candidate [121,141].

Recombinant VSV-based vaccines, as discussed for Ebola virus disease (Section 3.2), represent another major platform under investigation for filoviruses. An rVSVΔG vaccine expressing the MARV Musoke GP has demonstrated robust protective efficacy not only against the Musoke strain but also against the genetically distinct RAVN strain and the highly virulent Angola strain [125].

## 8. Dengue

Dengue, or dengue hemorrhagic fever (DHF), is a hemorrhagic fever disease caused by *Orthoflavivirus dengue* (dengue virus, DENV), which is mainly transmitted by mosquitoes of the genus *Aedes* (*Aedes aegypti* and *Aedes albopictus*) [1]. DENV is an enveloped, positive-sense RNA virus belonging to the *Flaviviridae* family, with a ~10.7 kb genome encoding three structural proteins (C, prM/M, E) and seven non-structural proteins involved in viral replication and immune evasion [142,143,144]. Among these, NS1 plays a key role in immune modulation, while NS2A and NS4B contribute to the organization of the viral replication complex [145,146]. DENV is classically classified into four antigenically distinct serotypes (DENV-1 to DENV-4), while a fifth serotype (DENV-5) has been reported but remains poorly characterized [147].

Dengue transmission remains most prevalent in Asia and South America; however, the geographical range of the disease has expanded substantially, with increasing reports in North America and Europe [148,149]. Travel-associated cases account for the majority of infections reported in non-endemic regions, including Europe, where climatic changes may further facilitate vector establishment and transmission [150].

Approximately 25% of infections are symptomatic, with a small proportion progressing to severe dengue [151]. Clinically, dengue typically presents as an acute febrile illness with headache, myalgia, arthralgia, and gastrointestinal symptoms, which may progress to vascular leakage, hemorrhagic manifestations, and organ dysfunction in severe cases [146,152]. The critical phase is characterized by increased vascular permeability and risk of shock, while recovery involves gradual fluid reabsorption and hematological normalization [153].

### 8.1. Therapeutic Approaches for Dengue Virus Infection

To date, no specific therapy for dengue has been approved; although several candidate molecules have been investigated, none have demonstrated significant clinical efficacy.

Although the antimalarial drug chloroquine showed activity against DENV serotype 2 in preclinical studies [154], it failed to demonstrate efficacy in clinical settings. In human trials, its administration did not result in measurable improvements in virological, immunological, or clinical outcomes, including reductions in viraemia or NS1 antigen levels or shortening of fever duration. Moreover, chloroquine did not prevent key hematological alterations associated with plasma leakage, such as thrombocytopenia and hematocrit elevation, and was associated with a higher incidence of gastrointestinal adverse events [155].

Other repurposed agents evaluated against DENV include lovastatin, as well as balapiravir and celgosivir, two antiviral compounds originally developed for hepatitis C virus infection. Despite acceptable safety profiles, these agents did not demonstrate meaningful clinical efficacy in dengue patients [156,157,158].

Among emerging therapeutic strategies, inhibitors targeting viral methyltransferase (MTase), helicase, protease, and NS4B, as well as entry/fusion blockers and the nucleoside analogue NITD008, have been investigated. Although these compounds demonstrated anti-DENV activity in vitro and in animal models, their progression to clinical evaluation has been limited by toxicity concerns and/or unfavorable pharmacokinetic profiles [156].

### 8.2. Vaccines for Dengue: Present Insights and Future Prospects

Developed by Sanofi Pasteur, Dengvaxia (CYD-TDV) is a live-attenuated, recombinant tetravalent dengue vaccine administered as a three-dose regimen. It represents the first licensed dengue vaccine worldwide, having received marketing authorization in the European Union in 2018 and approval from the U.S. Food and Drug Administration in 2019 [159]. CYD-TDV is based on a chimeric vaccine platform that employs the non-structural gene backbone of the yellow fever 17D vaccine strain, engineered to express DENV antigenic components and thereby induce immune responses against all four DENV serotypes (DENV-1–4) [160,161]. Although approved in multiple countries, its clinical use is restricted to individuals with confirmed prior dengue infection due to safety concerns observed in seronegative populations [162]. Moreover, Dengvaxia demonstrates limited protective efficacy in children younger than nine years of age and shows variable performance among the four dengue virus serotypes with respect to virologically confirmed symptomatic infection. Reported vaccine efficacy differs substantially by serotype, reaching 50.3% for DENV-1 and 42.3% for DENV-2, while higher levels of protection are observed against DENV-3 (74.0%) and DENV-4 (77.4%) [163]. The first public immunization program using Dengvaxia was implemented in the Philippines; however, it was permanently discontinued in 2019 following a major public health controversy that arose after reports of deaths among vaccinated children [164].

On 21 October 2025, the European Commission revoked the EU marketing authorization for Dengvaxia following a request from the marketing authorization holder, Sanofi Winthrop Industrie, which decided to permanently discontinue the product’s commercialization for economic reasons [159].

**Figure 7:**
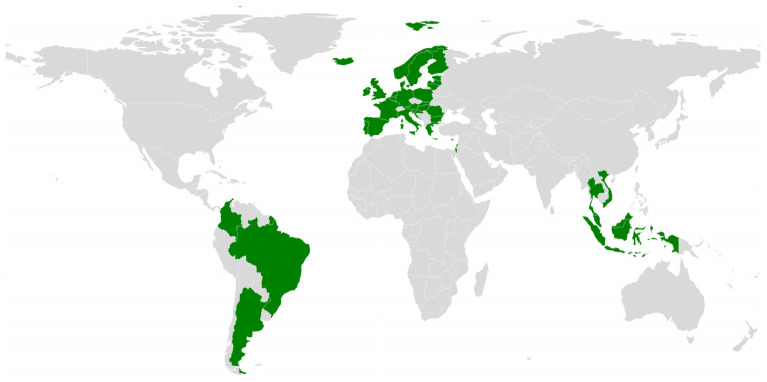
Global distribution of countries where the dengue vaccine Qdenga has received regulatory authorization. Countries highlighted in green indicate regions where the vaccine has been approved. Illustration created by the authors based on information reported in the literature [168]. This distribution reflects regional regulatory decisions and has implications for vaccine deployment, access, and large-scale implementation in endemic and non-endemic settings.

Qdenga (TAK-003), developed by Takeda Pharmaceuticals, is a second-generation, live-attenuated tetravalent dengue vaccine designed to provide protection against all four DENV serotypes. The vaccine consists of four chimeric DENV strains constructed using an attenuated DENV serotype 2 (DENV-2) backbone. As with other live-attenuated and viral platform-based vaccines discussed earlier (Section 3.2), this design aims to elicit broad immune responses across multiple serotypes [160,165]. The vaccination regimen consists of two doses administered with a three-month interval between injections [166]. Following its initial regulatory approval in Indonesia in 2022, Qdenga has subsequently received authorization in 41 countries, with approximately 18.6 million doses supplied across 11 dengue-endemic regions by September 2025 (Figure 7). In addition, the vaccine has been included in the WHO List of Prequalified Vaccines, highlighting its validated quality, safety, and suitability for incorporation into large-scale public immunization programs aimed at mitigating the global burden of dengue [167,168].

In November 2025, Takeda Pharmaceuticals reported the completion of the 7-year pivotal Phase 3 Tetravalent Immunization against Dengue Efficacy Study (TIDES) evaluating Qdenga. The results confirmed a favorable benefit–risk profile and demonstrated that the approved two-dose regimen provides durable protection against dengue, consistent with its authorized use in multiple countries. At 4.5 years, vaccine efficacy (VE) against virologically confirmed dengue was 61.2% (95% CI: 56.0–65.8). An exploratory booster dose administered at 4.5 years resulted in a modest increase in VE to 74.3% (95% CI: 66.7–80.1) after two additional years. Protection against dengue-related hospitalization remained high, with VE of 84.1% (95% CI: 77.8–88.6) at 4.5 years and 90.6% (95% CI: 78.9–95.8) following the booster. Efficacy was observed across all four dengue virus serotypes through seven years of follow-up, and no new safety signals were identified after booster administration [167].

#### Butantan-DV (TV003)

The live-attenuated tetravalent dengue vaccine TV003 was designed by the National Institute of Allergy and Infectious Diseases (NIAID; Bethesda, MD, USA). Unlike other dengue vaccines described above, this candidate comprises four genetically distinct attenuated dengue virus components. Attenuation of the DENV-1, DENV-3, and DENV-4 strains was achieved through targeted deletions at the 3′ terminal region of their genomes. For the DENV-2 component, a chimeric virus (DENV4/2) was engineered by inserting the prM–E coding sequence of DENV-2 into an attenuated DENV-4 backbone. Unlike Dengvaxia and Qdenga, which employ a uniform viral backbone strategy, TV003 adopts a multivalent attenuation approach to promote equivalent immune responses across all four dengue serotypes [169,170,171,172].

Technology transfer agreements have enabled the licensing of TV003 to multiple manufacturers worldwide, including the Butantan Institute (Brazil), a leading global biomedical research institution. In response to the country’s substantial dengue burden (more than 20 million cases since 2000; 8.1 million probable cases between January 2024 and November 2025 alone), the Butantan Institute conducted a large-scale phase III clinical study between 2016 and 2024, enrolling more than 16,000 participants across 14 Brazilian states to evaluate the safety and efficacy of the locally produced Butantan-DV vaccine [173,174].

Preliminary findings from the phase III clinical trial, based on a two-year follow-up, demonstrated that the Butantan-DV vaccine achieved an overall efficacy of 79.6% against virologically confirmed dengue, with consistent protection observed across age strata and baseline serostatus. When stratified by prior dengue exposure, vaccine efficacy reached 89% in seropositive individuals and 74% in seronegative individuals. Serotype-specific analyses showed particularly high efficacy against DENV-1 and DENV-2, with higher protection among seropositive participants (97% for DENV-1 and 84% for DENV-2) compared with seronegative participants (86% for DENV-1 and 58% for DENV-2). Across the study population, efficacy estimates of 89.5% for DENV-1 and 69.6% for DENV-2 further confirmed the vaccine’s strong serotype-specific performance. The vaccine was generally well tolerated; although adverse events were reported more frequently in vaccine recipients than in placebo recipients within 21 days following vaccination, no serious safety signals were identified during the two-year observation period [173,175,176].

Final trial analyses demonstrated that, among participants aged 12–59 years, the vaccine achieved an overall efficacy of 74.7%. Protection was substantially higher against severe clinical outcomes, reaching 91.6% for severe dengue and dengue with warning signs, while complete protection (100% efficacy) was observed against dengue-related hospitalizations [174].

In December 2025, Brazil’s national regulatory authority, Anvisa, formally approved the single-dose Butantan-DV vaccine, developed by the Butantan Institute through a partnership coordinated by the Brazilian Ministry of Health and the Chinese manufacturer WuXi Vaccines. Administration of the vaccine is planned to begin in 2026 through Brazil’s public healthcare system, with doses provided free of charge [177].

Beyond the three licensed dengue vaccines, multiple strategies are being pursued to develop next-generation formulations with improved efficacy and safety profiles. The main approaches currently under investigation are summarized in Table 3:

Future dengue vaccine research is progressing toward broader and more durable protection against all four virus serotypes, with the aim of simplifying immunization strategies and minimizing partial immunity. Efforts are also focused on improving safety and effectiveness, particularly in individuals without prior dengue exposure, through a better understanding of vaccine-associated risks. In parallel, the exploration of innovative platforms, including mRNA-based, viral vector, and nanoparticle vaccines, offers new opportunities to optimize immune responses and accelerate vaccine development. Combination vaccine strategies targeting dengue alongside other mosquito-borne diseases may further enhance future prevention efforts [182].

## 9. Yellow Fever

Yellow fever (YF) is a hemorrhagic disease caused by *Orthoflavivirus flavi* (yellow fever virus, YFV), a member of the *Flaviviridae* family (Table S1). Transmission occurs via blood-feeding mosquitoes, primarily *Haemagogus* and *Sabethes* species in sylvatic cycles and *Aedes* species in urban settings [183,184].

**Figure 8:**
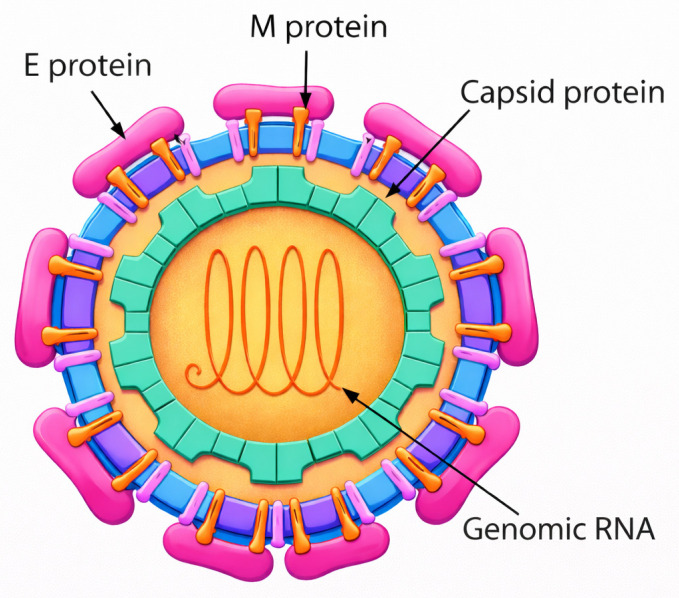
Schematic representation of the structural organization of yellow fever virus (YFV), illustrating the envelope (E) protein, membrane (M) protein, capsid protein, and the genomic RNA. Adapted from ViralZone [188]. These structural components are directly relevant for vaccine antigen selection and antiviral targeting and may guide formulation and immunogen design strategies.

YFV is an enveloped, positive-sense single-stranded RNA virus (~11 kb) encoding a single polyprotein that is cleaved into three structural proteins (C, prM/M, E) and seven non-structural proteins involved in viral replication and immune evasion (Figure 8) [185,186,187].

Yellow fever remains endemic in sub-Saharan Africa and tropical regions of South and Central America, where periodic outbreaks continue to occur [189,190]. Several large outbreaks have been reported in recent decades, including in Africa and Brazil, highlighting the persistent epidemic potential of YFV and its capacity for geographic expansion beyond traditional endemic zones [191,192,193,194,195,196,197]. More recent events, such as the outbreak in Colombia in 2024–2025, further underscore ongoing transmission risks, particularly in rural and sylvatic settings [198].

Most infections are asymptomatic or mild; however, symptomatic disease typically presents after an incubation period of 3–7 days with acute febrile illness, including headache, myalgia, and gastrointestinal symptoms. While many patients recover, a subset progresses to severe disease characterized by jaundice, hemorrhagic manifestations, shock, and multi-organ dysfunction. Case fatality rates in severe cases range from 30% to 60%, and survivors generally develop long-lasting immunity [199,200,201,202].

### 9.1. Therapeutic Approaches

**Figure 9:**
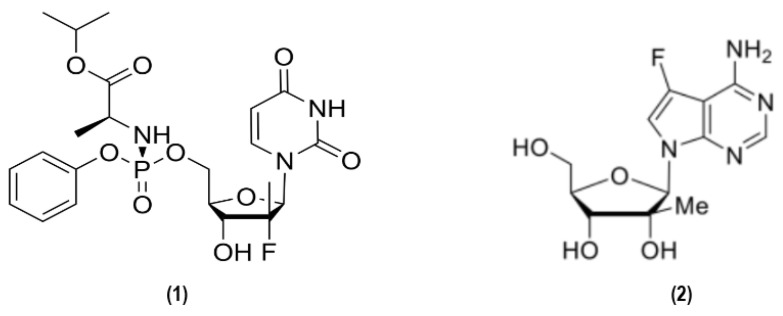
Chemical structures of sofosbuvir (**1**) and DFA (**2**).

At present, no antiviral agents have been approved for the treatment of yellow fever virus infection, and patient management remains limited to supportive and palliative care. Nevertheless, considerable efforts have been undertaken to identify effective therapeutic options. One promising approach involves the repurposing or development of nucleoside analogues targeting YFV replication. Compounds such as sofosbuvir (Figure 9), remdesivir and galidesivir (see Table 1) have demonstrated antiviral activity against YFV in vitro and in animal models; however, further investigation is required to confirm their efficacy [203,204,205]. In a recent study, Le Cher et al. reported that 7-deaza-7-fluoro-2′-C-methyladenosine (DFA; Figure 9) markedly reduced liver pathology and viral load in mice infected with either a vaccine strain or a clinical isolate that models severe human disease, thereby supporting the continued preclinical development of DFA [206].

Monoclonal antibody-based therapies have also been explored as a targeted antiviral strategy. As outlined for other VHFs (Section 3.1), such antibodies provide high specificity and virus-neutralizing activity, with potential applications in both prophylactic and therapeutic settings [207].

MBL-YFV-01 is a fully human mAb that has been optimized and evaluated by Mabloc, a biotechnology company specializing in the isolation and development of monoclonal antibodies. This candidate represents a novel therapeutic option for yellow fever virus infection, including potential use in emergency post-exposure settings. In a non-human primate study, administration of a single 50 mg/kg dose of MBL-YFV-01 completely suppressed viremia and provided full protection against severe disease and mortality in treated animals [208]. These findings were subsequently supported by additional primate research, which demonstrated that multiple dosing regimens were effective in preventing death and further showed the prophylactic potential of the antibody [207].

Recently, Mabloc announced a strategic collaboration with the Butantan Institute to advance the co-development and manufacturing of MBL-YFV-01. This program aims to support the development of a disease-specific therapeutic candidate for yellow fever [209].

### 9.2. Overview of Yellow Fever Vaccines

In 1928, the South African physician Max Theiler initiated yellow fever vaccine development by serially passaging the Asibi virus strain, originally isolated by Stokes, in chick embryos and mouse tissues. After 176 passages, the resulting live-attenuated strain, designated 17D, induced protective neutralizing antibodies in NHPs without causing visceral or neurological damage and conferred protection against lethal challenge. Based on these findings, 17D progressed to human use, with the first vaccinations administered in 1937 during a yellow fever outbreak in Minas Gerais, Brazil [184,194].

WHO guidelines indicate that the only licensed yellow fever vaccine in current use is the live-attenuated 17D strain. All vaccines administered today are derived from one of three closely related 17D substrains—17D-204, 17DD, and 17D-213—which differ in passage history and seed lot derivation. The 17D-204 substrain originates from passage 204 of the Asibi strain, while 17DD was independently derived at passage 195 following serial passage in chicken tissue. The 17D-213 substrain was later generated from 17D-204 under the coordination of the WHO by the Robert Koch Institute, with vaccine seed lots produced at passages 237–238 [210].

Currently, yellow fever vaccines are manufactured by six producers worldwide, each using one of these three substrains. The 17D-204 vaccine is produced by Sanofi Pasteur and marketed as YF-VAX^®^ in the United States and as Stamaril^®^ by Sanofi Pasteur France for international distribution; it is also manufactured in China by the China National Biotec Group (CNBG) under the name Tiantan^®^. The 17DD vaccine is produced in Brazil by Bio-Manguinhos/Fiocruz, while Senegal manufactures a 17D-204–based vaccine through the Institut Pasteur de Dakar. In the Russian Federation, the 17D-213 vaccine is produced by the Chumakov Federal Scientific Center and marketed as SinSaVac™. Together, these vaccines support global immunization efforts coordinated by WHO and UNICEF [211].

Licensed yellow fever vaccines have a well-established record of safety and efficacy, providing robust and long-lasting protective immunity. However, their production relies on manufacturing technologies developed several decades ago, which depend on pathogen-free embryonated eggs for large-scale vaccine generation. This reliance, together with recent yellow fever outbreaks, has contributed to vaccine shortages resulting from limited stockpiles and the inability of complex, labor-intensive manufacturing processes to rapidly respond to increased global demand. As discussed for other vaccine platforms (Section 3.2), improving manufacturing flexibility and scalability remains a key objective for next-generation vaccines. Therefore, there is a need for the development of novel yellow fever vaccines that maintain or surpass the safety and efficacy profiles of existing vaccines while offering improved scalability and shorter production timelines [212,213,214].

Yellow fever vaccine candidates under development include mRNA vaccines, virus-like particles (VLPs), plant-derived subunit vaccines, plasmid-vectored DNA constructs, recombinant vaccinia-based vaccines, inactivated vaccines, and live-attenuated vaccines incorporating synonymous mutations. These approaches reflect broader trends in vaccine platform development described earlier (Section 3.2). Most candidates remain in preclinical development, with only two having progressed to Phase I clinical trials [210]. One such candidate, XRX-001 (PnuVax), is an inactivated derivative of the live-attenuated 17D strain, produced in Vero cells and adsorbed to an alum adjuvant. This formulation is intended to improve the safety profile, particularly for individuals with egg or gelatin allergies, infants, pregnant women, older adults, and immunocompromised populations. As a non-replicating vaccine, XRX-001 reduces the risk of viscerotropic disease, a rare but severe adverse reaction resembling wild-type yellow fever. In Phase I studies, neutralizing antibodies were detected in 100% of high-dose recipients and 88% of low-dose recipients [215,216].

MVA-BN YF is a nonreplicating viral vector vaccine developed by Bavarian Nordic and constructed by inserting gene sequences encoding the yellow fever virus preM and E proteins, derived from the reference viral genome (NCBI Accession No. NC_002031), into the MVA-BN^®^ platform [217]. The recombinant virus was propagated in primary chicken embryo fibroblast cells under serum-free culture conditions. For formulation, MVA-BN YF was combined with Montanide™ ISA 720 VG (SEPPIC S.A., Paris, France), a non-mineral oil–based adjuvant, to form a stable emulsion [218]. A preclinical study showed that the vaccine candidate fully protected hamsters against viral challenge and indicated that formulation with ISA 720 elicited a robust immune response following a single immunization [219]. A Phase I clinical trial conducted between 2016 and 2018 evaluated the safety, reactogenicity, and immunogenicity of the MVA-BN yellow fever vaccine, with and without the Montanide ISA-720 adjuvant, in healthy volunteers aged 18–45 years; however, the study results have not yet been reported [220].

To date, no publicly available information has been reported regarding the progression of the XRX-001 and MVA-BN YF vaccines into Phase II clinical trials [213].

## 10. Other VHFs

Other VHFs share several common features with those discussed above in terms of therapeutic approaches and immunoprophylactic strategies. Rather than addressing each individually in detail, the main aspects of therapy and immunoprophylaxis are summarized in Table 4 to provide a concise overview.

**Table 4:** Therapy and immunoprophylaxis of selected viral hemorrhagic fevers.

Disease/ Virus/Family	Therapeutic Approach	Refs.	Immunoprophylaxis	Refs.
Kyasanur Forest disease (KFD) *Orthoflavivirus* *kyasanurense* (Kyasanur Forest Disease virus, KFDV) *Flaviviridae*	No specific approved therapy; repurposed drugs (e.g., broad-spectrum antivirals favipiravir, sofosbuvir, and the anthelmintic niclosamide) have shown in vitro activity against multiple flaviviruses; They have not been evaluated for KFD.	[221,222,223]	Formalin-inactivated KFDV vaccine (India, since 1960s): moderate efficacy (~62% after two doses, ~83% with booster); immunity wanes within 1 year; annual boosters required; production constraints and vaccine hesitancy limit coverage. Next-generation KFD vaccines: recombinant subunit, viral-vector, and mRNA platforms under investigation; VSV-based vaccine expressing KFDV M/E proteins protective in mice and NHPs; multi-epitope subunit vaccine shows strong predicted B- and T-cell responses (in silico).	[222,224,225,226,227]
Omsk hemorrhagic fever (OHF) *Orthoflavivirus omskense* (Omsk hemorrhagic fever virus, OHFV) *Flaviviridae*	No specific approved therapy. Ribavirin (Figure 10): nucleoside analogue; limited efficacy (in vitro inhibition, partial protection in mice, reduced disease duration in rabbits); toxicity reported at high doses. Larifan (dsRNA): bacteriophage-derived immunomodulator; antiviral activity against OHFV in vivo (~65% survival in mice), but limited in vitro efficacy.	[228,229]	No approved human vaccines or clinical trials to date. Epitope-based vaccine (in silico): predicted antigenicity, low toxicity, and strong TLR4 binding, suggesting potential to induce innate and adaptive immune responses.	[230]
Lassa fever (LF) *Mammarenavirus* *lassaense* (Lassa virus, LASV) *Arenaviridae*	No approved antivirals. Ribavirin: benefit mainly with early IV or oral administration; reduced efficacy with delayed treatment. Favipiravir: dose-dependent protection in guinea pig and NHP models; clinical efficacy in humans remains uncertain.	[231,232,233]	rVSVΔG-LASV-GPC (VSV-based): Phase I trials showed favorable safety and cross-lineage antibody responses; Phase II studies ongoing in West Africa. EBS-LASV (VesiculoVax platform): VSV-based vaccine undergoing Phase I trials in Ghana. INO-4500 (DNA vaccine): LASV GPC-encoding vaccine; Phase I trials show acceptable safety and immunogenicity; protective efficacy in animal models. MV-LASV (measles-vectored): induces humoral and T-cell responses; Phase I trials demonstrate acceptable safety and immunogenicity.	[231,234,235,236,237,238,239]
Argentine hemorrhagic fever (AHF) *Mammarenavirus juninense* (Junin virus, JUNV) *Arenaviridae*	No specific approved antiviral therapy. Ribavirin: evaluated intravenously for AHF but showed no significant mortality reduction. Favipiravir: enhanced antiviral activity against JUNV when combined with ribavirin. Fusion inhibitors: antiviral activity against arenaviruses including JUNV; therapeutic efficacy remains to be established.	[240,241,242,243]	Candid#1 vaccine: live-attenuated JUNV vaccine licensed in Argentina. Recombinant baculovirus vaccine (JUNV GP1/GP2): induces neutralizing antibodies and partial protection in experimental studies.	[244,245]
Crimean–Congo hemorrhagic fever (CCHF) *Orthonairovirus* *haemorrhagiae* (Crimean–Congo hemorrhagic fever virus, CCHFV) *Nairoviridae*	Ribavirin: widely used for CCHF treatment; clinical benefit remains debated due to limited randomized trials; administered orally or intravenously. In severe cases, combination with high-dose methylprednisolone may improve outcomes. Favipiravir: RNA polymerase inhibitor; demonstrated antiviral activity and protection in CCHFV animal models, including late treatment; shows in vitro synergy with ribavirin. A Phase I/II trial is evaluating ribavirin–favipiravir combination therapy. Other therapeutic approaches: nucleoside analogues (e.g., 2′-deoxy-2′-fluorocytidine), viral protease inhibitors (e.g., UbV-CC4), and monoclonal antibodies show preclinical antiviral activity; further validation required.	[246,247,248]	ChAdOx2 CCHF vaccine: replication-deficient adenoviral vector vaccine; clinical trial initiated in 2023 to evaluate safety and immunogenicity. HDT-321 vaccine: self-amplifying RNA vaccine formulated with nanoparticle delivery system; Phase I clinical trial initiated in 2025 to assess safety and immunogenicity. N-pVAX1 vaccine: DNA vaccine targeting CCHFV antigens; Phase I trial initiated in 2024 evaluating safety and immunogenicity following intramuscular administration with electroporation. MVA-CCHF vaccine: modified vaccinia Ankara-vectored candidate; Phase I trial completed evaluating safety and immunogenicity after two intramuscular doses.	[249,250,251,252]
Hantavirus hemorrhagic fever with renal syndrome (HFRS) *Orthohantavirus*: *-hantanense* (Hantaan virus, HTNV) *-seoulense* (Seoul hantavirus, SEOV) *-puumalaense* (Puumala hantavirus) -*dobravaense* (Dobrava-Belgrade hantavirus) *Hantaviridae*	No specific approved therapy. Ribavirin: early administration reduces morbidity and mortality in HFRS caused by Seoul and Hantaan viruses; limited benefit with delayed treatment and no clear efficacy against Puumala virus. Lactoferrin: innate immune glycoprotein; partial protection against HFRS in mouse models, with complete inhibition of viral focus formation when combined with ribavirin. Monoclonal antibodies (ADI-42898/ADI-65534): broadly neutralizing antibodies targeting the Gn/Gc glycoprotein complex; protection demonstrated in rodent models, including post-exposure efficacy.	[253,254,255,256]	Hantavax (inactivated Hantaan virus vaccine): licensed in South Korea; high seroconversion rates but variable neutralizing antibody responses and debated protective efficacy. Inactivated orthohantavirus vaccines (China): monovalent and bivalent HTNV/SEOV vaccines used in national immunization programs; associated with reduced HFRS incidence in endemic regions. Next-generation orthohantavirus vaccines: viral-vector, virus-like particle, subunit, and nucleic-acid platforms under development; challenges include limited breadth of protection and manufacturing constraints.	[255,257,258]
Rift Valley fever (RVF) *Phlebovirus riftense* (Rift Valley Fever virus, RVFV) *Phenuiviridae*	No specific approved therapy. Ribavirin/Favipiravir: antiviral activity demonstrated in rodent models; clinical use of ribavirin limited by safety concerns, including neurological complications reported during the 2000 Saudi Arabia outbreak. 4′-Fluorouridine (4′-FlU; EIDD-2749) (Figure 10): uridine-analog nucleoside showing antiviral activity against RVFV in vitro; oral administration provided full protection in murine models, including post-exposure treatment.	[259,260,261]	Formalin-inactivated RVFV vaccines (NDBR-103; TSI-GSD-200): evaluated in humans and used in high-risk groups; induce neutralizing antibodies but limited by safety concerns and waning immunity requiring boosters. MP-12 vaccine: live-attenuated RVFV strain; demonstrated immunogenicity and protection in preclinical models and Phase I/II trials. ChAdOx1 RVF vaccine: replication-deficient adenoviral vector encoding RVFV Gn/Gc glycoproteins; protective in livestock models and safe and immunogenic in Phase I trials. Additional vaccine platforms: recombinant, DNA, subunit, and replicon vaccines under preclinical or early-stage development.	[262,263,264,265]

**Figure 10:**
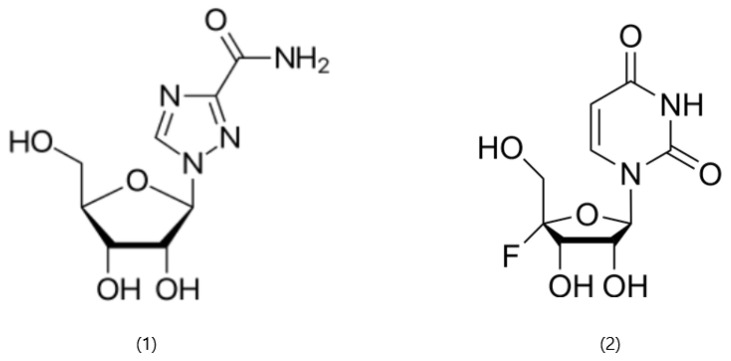
Chemical structures of Ribavirin (**1**) and 4′-Fluorouridine (**2**).

## 11. Pharmaceutical and Formulation Considerations for VHF Therapeutics and Vaccines

The development of effective countermeasures against viral hemorrhagic fevers (VHFs) requires not only the identification of antiviral agents and vaccine platforms but also careful consideration of pharmaceutical factors including formulation design, delivery strategies, pharmacokinetics, stability, and large-scale manufacturing. Many of the currently approved or investigational countermeasures for VHFs are biologics—particularly mAbs and viral-vector vaccines—which present specific pharmaceutical challenges related to formulation stability, storage conditions, and delivery requirements [266].

Monoclonal antibody therapeutics represent the most clinically advanced pathogen-targeted treatments currently available for filovirus infections. As discussed in Section 3.1, two antibody-based products—ansuvimab (Ebanga) and the triple-antibody cocktail atoltivimab/maftivimab/odesivimab (REGN-EB3, marketed as Inmazeb)—have received regulatory approval for the treatment of Ebola virus disease. Because no pathogen-targeted therapeutics have yet been approved for other filoviruses, these agents provide the clearest currently approved examples for discussing the pharmaceutical characteristics of biologic antivirals developed for VHFs. Beyond their antiviral activity, these biologics illustrate several important pharmaceutics considerations including formulation stability, parenteral delivery requirements, pharmacokinetic behavior, and cold-chain storage constraints that influence the practical deployment of antibody-based therapies during outbreak responses [267].

From a pharmaceutical perspective, monoclonal antibody therapeutics require carefully optimized formulations to minimize protein aggregation, denaturation, or chemical degradation during storage and administration. Antibody formulations typically contain buffering systems—such as histidine, phosphate, or citrate buffers—to maintain appropriate pH conditions and preserve protein structure. Stabilizing excipients, including disaccharides such as sucrose or trehalose, are commonly incorporated to protect proteins during freezing or drying processes, while non-ionic surfactants such as polysorbate-20 or polysorbate-80 help reduce interfacial stress and prevent surface-induced aggregation. These excipients play an essential role in maintaining the conformational and colloidal stability of therapeutic antibodies throughout manufacturing, storage, and administration. Depending on the formulation strategy, antibody-based therapeutics may be supplied either as sterile liquid solutions or as lyophilized powders that require reconstitution prior to administration in order to enhance long-term stability. Because antibodies are large protein molecules that are susceptible to enzymatic degradation in the gastrointestinal tract and exhibit poor epithelial permeability, they must be administered parenterally, most commonly by intravenous infusion. These formulation and delivery considerations are particularly important for therapeutics intended for use during outbreak responses, where stability during transport, ease of preparation, and compatibility with field-based clinical infrastructure are critical for successful deployment [268,269,270].

Pharmacokinetic characteristics represent another important pharmaceutical consideration. Monoclonal antibodies generally exhibit relatively long elimination half-lives, typically on the order of two to three weeks, due to neonatal Fc receptor (FcRn)-mediated recycling of IgG molecules. This mechanism protects antibodies from lysosomal degradation and prolongs systemic exposure, allowing sustained therapeutic concentrations following a single administration—an important advantage in outbreak settings where repeated dosing may be logistically difficult [271].

Cold-chain requirements represent a major logistical and pharmaceutical challenge for biologic countermeasures targeting VHFs. Many biologic therapeutics and viral-vector vaccines must be stored under refrigerated or frozen conditions to maintain structural integrity and biological activity. Maintaining these storage conditions can be particularly challenging in remote or resource-limited regions where VHF outbreaks frequently occur. Deviations from recommended storage temperatures during transport or field deployment may compromise product stability and reduce therapeutic effectiveness. For example, the rVSV-based Ebola vaccine (Ervebo) requires long-term storage at very low temperatures, typically between −80 °C and −60 °C, with only limited storage permitted at refrigerated temperatures after thawing. These temperature requirements illustrate the logistical challenges associated with distributing biologic countermeasures during emergency outbreak responses [272,273].

In addition to therapeutic antibodies, vaccine technologies for VHFs present distinct formulation challenges. Several licensed or investigational vaccines rely on viral-vector platforms, including rVSV vectors and adenovirus-based vectors. The formulation of these vaccines must preserve viral infectivity and antigen expression capacity while maintaining stability during storage and distribution. Achieving this requires carefully optimized buffer systems, cryoprotectants, and stabilizing excipients that protect viral particles against degradation during freezing, thawing, and transport. Formulation parameters such as pH, ionic strength, and stabilizer composition therefore play critical roles in ensuring vaccine potency throughout the supply chain [79].

Another important pharmaceutical consideration in the development of medical countermeasures for VHFs is large-scale manufacturing capacity. Biologic therapeutics such as monoclonal antibodies and viral-vector vaccines are typically produced using complex mammalian cell-culture systems followed by multi-step purification and rigorous quality-control procedures. These manufacturing requirements may limit the rapid scale-up of production during epidemic emergencies, particularly in regions with limited biomanufacturing infrastructure [274].

Manufacturing constraints are also evident in the production of certain live-attenuated vaccines used against hemorrhagic viruses. For example, the widely used yellow fever 17D vaccine is traditionally produced using a seed-lot system in embryonated chicken eggs, a technology originally established in the mid-twentieth century and still used today for large-scale vaccine manufacturing. In this process, primary seed viruses generate secondary seed stocks that are subsequently used to produce vaccine batches intended for human immunization. The resulting vaccine is typically supplied as a freeze-dried formulation that is reconstituted prior to administration. International regulatory guidelines require that each dose contains a minimum viral potency (expressed in international units), while long-term stability requirements—often at least three years—necessitate robust formulation strategies to preserve vaccine viability during storage and distribution [210].

Despite improvements in production yields over time, egg-based manufacturing remains constrained by the availability of specific pathogen-free eggs, which can represent a limiting factor in large-scale vaccine production. For this reason, alternative manufacturing approaches are actively being explored, including adaptation of the 17D virus to continuous cell substrates such as Vero cells. Experimental studies have demonstrated that vaccine candidates derived from these cell-culture systems can maintain favorable safety profiles and strong immunogenicity in preclinical models, while potentially enabling more scalable and standardized manufacturing processes. These efforts highlight the importance of developing flexible production platforms capable of supporting rapid vaccine supply during epidemic outbreaks [275].

Overall, advances in manufacturing technologies—including optimized cell-culture platforms, improved purification strategies, and scalable production systems—are expected to play an essential role in ensuring the timely availability of vaccines and biologic therapeutics during outbreaks of viral hemorrhagic fevers.

While Table 1 summarizes the clinical development status and antiviral mechanisms of investigational therapeutics for Ebola virus disease, Table 5 comparatively summarizes the pharmaceutical characteristics of selected approved countermeasures, including both therapeutics and vaccines. The table highlights key formulation parameters such as dosage form, route of administration, pharmacokinetic behavior, and storage requirements that influence the practical deployment of these medical countermeasures in outbreak settings. The information presented in Table 5 is based on regulatory product documentation and published literature describing the development, formulation, and pharmacological characteristics of approved therapeutics and vaccines for VHFs [55,79,210,276].

The comparative overview illustrates that mAbs therapeutics currently represent the most clinically advanced pathogen-targeted countermeasures for EVD, combining high target specificity with prolonged systemic exposure. In contrast, vaccine platforms provide the most effective strategy for long-term outbreak prevention and population-level protection. However, both therapeutic antibodies and viral-vector vaccines present important pharmaceutical and practical challenges, including cold-chain dependence, complex manufacturing processes, regulatory heterogeneity across regions, and administration requirements that may limit rapid deployment, particularly in resource-limited settings. Addressing these logistical and pharmaceutical barriers—through improved formulation stability, alternative delivery technologies, scalable production strategies, and streamlined regulatory pathways—remains a central objective in the development of next-generation therapeutics and vaccines for VHFs.

## 12. Conclusions

VHFs continue to pose a substantial global health threat. Although many of these infections remain concentrated in endemic regions of Africa, Asia, and the Americas, their wide geographic distribution, together with increasing international travel and tourism, facilitates the importation of cases into non-endemic areas and raises the risk of secondary transmission where competent vectors or favorable conditions exist. As a result, VHFs can no longer be regarded as strictly local diseases.

These infections are associated with high mortality rates and a disproportionate social and economic burden. In addition to severe acute illness and frequent fatal outcomes, outbreaks strain healthcare systems, disrupt essential services, and leave long-term physical and psychological consequences among survivors and affected communities. Such impacts strongly justify the urgent need for effective therapeutic options and preventive strategies.

At present, specific therapeutic means for VHFs remain limited. Supportive care is still the cornerstone of treatment for most infections, whereas pathogen-targeted therapies are available only for a restricted number of diseases. Among the different therapeutic approaches explored to date, monoclonal antibodies have emerged as the most successful. This is exemplified by the approval of two mAb-based treatments for Ebola virus disease, and by the large and growing body of literature investigating monoclonal antibodies for other VHFs.

In parallel, numerous antiviral compounds—including nucleoside analogues and other broad-spectrum antivirals—have been evaluated in preclinical studies and, in some cases, in clinical trials conducted during outbreak situations. While many investigational therapeutics have shown encouraging activity in NHP models, the transition from experimental efficacy to consistent clinical benefit remains uncertain. Factors such as variability in disease presentation, the timing of therapeutic intervention, and the operational challenges of conducting controlled studies during epidemics continue to complicate the clinical translation of promising candidates. Despite encouraging activity observed in experimental systems, the clinical development of many antiviral candidates has been hindered by variability in efficacy across different VHFs, safety considerations, and the critical influence of treatment timing during acute infection. In addition, the logistical and methodological constraints associated with conducting controlled studies during epidemic outbreaks have complicated the evaluation of therapeutic benefit and slowed regulatory approval. As a result, no small-molecule antiviral has yet received universal regulatory approval for the treatment of VHFs. Comparative evaluation of current therapeutic strategies indicates that monoclonal antibody-based approaches have achieved the most consistent clinical success for filovirus infections, whereas small-molecule antivirals and host-targeted strategies remain under active investigation for broader application across multiple VHFs.

The situation is comparatively more encouraging for vaccines. Licensed vaccines are available for a small number of VHFs, such as Ebola, yellow fever, and dengue, and multiple investigational vaccines using diverse technological platforms are advancing through clinical development. Nevertheless, vaccine availability remains limited, and many VHFs still lack effective preventive options.

Future progress in the field will likely depend on coordinated advances in both therapeutics and vaccines, supported by improved diagnostics, robust outbreak-ready clinical trial designs, and a One Health perspective that integrates human, animal, and environmental surveillance. Together, these efforts are essential to reduce the global impact of VHFs and to better prepare for future outbreaks.

## Figures and Tables

**Table 1:** Experimental therapeutic agents investigated for EVD.

Drug Name	Class and Structural Considerations	Comment	Pharmaceutical and Practical Deployment Considerations	Refs.
Monoclonal antibody-based therapeutics				
ZMapp	Monoclonal antibodies (mAbs) (cocktail of three mAbs: 2G4, 4G7 and 13C6)	First used in 2014 to treat two American missionaries with severe Ebola in Liberia, ZMapp led to clinical improvement and reduced viremia. The PREVAIL II RCT (2015) showed higher survival with ZMapp (78%) than standard care (63%), though not statistically significant. Later, ZMapp served as a control in the PALM trial and was discontinued due to higher mortality (50%) compared with mAb114 and REGN-EB3 (≈35%).	Intravenous administration; cold-chain required; complex biomanufacturing; limited manufacturing scalability	[39,40,41]
MBP 431	Cocktail of two mAbs: ADI-15878 and ADI-23774	In a recent trial, a single 5 mg/kg intramuscular dose of MBP431 provided strong protective efficacy, far lower than the FDA-approved doses for mAb114 (50 mg/kg) and REGN-EB3 (150 mg/kg). Future studies are needed to determine whether combining lower MBP431 doses with small-molecule antivirals like Remdesivir could further enhance protection in severe cases through improved biodistribution.	Parenteral monoclonal antibody cocktail; cold-chain dependent; high manufacturing complexity; manufacturing scalability not yet established	[42]
Nucleoside analogue antivirals (RNA polymerase inhibitors)				
Remdesivir (GS-5734)	Broad spectrum antiviral agent; adenosine analogue nucleotide prodrug 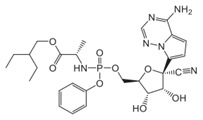	Remdesivir is a nucleoside analogue inhibitor of viral RNA-dependent RNA polymerase originally developed for broad-spectrum antiviral activity against RNA viruses, including EBOV. It is metabolized to the active compound GS-441524. In the PALM trial, remdesivir achieved a survival rate of approximately 47% in Ebola virus disease patients, showing lower efficacy than antibody-based therapies. Combination strategies with monoclonal antibodies have been proposed to improve clinical outcomes and reduce viral persistence.	intravenous formulation; hospital-based use; limited field feasibility of use; manufacturing scalability feasible but resource-demanding	[43,44,45]
Favipiravir (T-705)	broad-spectrum antiviral ribonucleoside analogue; pyrazineccarboxamide derivative 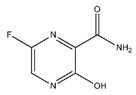	Favipiravir is a broad-spectrum nucleoside analogue inhibitor of viral RNA-dependent RNA polymerase. In the JIKI trial (2014–2015) conducted in Guinea, favipiravir showed limited efficacy in Ebola virus disease patients, partly due to suboptimal plasma concentrations. These findings suggest that higher dosing regimens may be required to achieve therapeutic exposure.	oral formulation; variable pharmacokinetics; dose optimization required; favorable manufacturing scalability	[46]
Galidesivir (BCX4430)	broad-spectrum antiviral; adenosine nucleoside analog 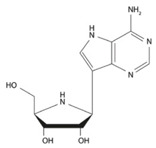	Galidesivir is a nucleoside analogue inhibitor of viral RNA-dependent RNA polymerase that has demonstrated potent activity against EBOV in preclinical studies. In animal models, treatment provided high survival rates following lethal viral challenge, including complete protection in non-human primates when administered shortly after infection and reduced survival when treatment initiation was delayed. These findings highlight the promising antiviral activity of galidesivir in preclinical models, although further studies are required to determine its clinical efficacy in humans.	parenteral administration; limited clinical data; formulation under evaluation; manufacturing scalability not yet established	[47,48]
Obeldesivir (GS-5245)	Antiviral agent 5′-isobutyryl ester prodrug of GS-441524, the major circulating nucleoside metabolite of remdesivir 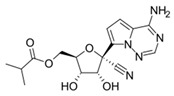	A recent study reported that obeldesivir provided substantial post-exposure protection in nonhuman primate (NHP) models, achieving 80% survival in *Cynomolgus macaques* and 100% survival in rhesus macaques when administered 24 h after EBOV challenge. Treatment was also associated with marked reductions, and in many cases complete clearance, of EBOV RNA in immune-privileged tissues, including the brain, spinal cord, ocular and testicular tissues. These findings highlight the promising antiviral activity of obeldesivir in preclinical models, although its clinical efficacy in humans remains to be established.	oral prodrug; improved bioavailability relative to remdesivir; early clinical development; manufacturing scalability likely favorable	[49]
RNA-targeting therapeutics				
TKM-130803	small interfering RNAs (siRNA)-based lipid nanoparticle	A phase 2 trial of TKM-130803, an siRNA-based lipid nanoparticle therapy targeting EBOV polymerase and VP35, was conducted in Sierra Leone in 2015. The earlier formulation (TKM-100802) was ineffective against the West African strain, prompting redesign. In the trial, 14 patients received seven doses, but no survival benefit was observed, leading to early termination and the conclusion that advanced Ebola was unresponsive to TKM-130803.	siRNA–lipid nanoparticle delivery; stability and delivery challenges; intravenous administration; complex manufacturing with limited scalability	[50,51,52]
Other antiviral agents				
Brincidofovir	Antiviral agent targeting viral DNA polymerase 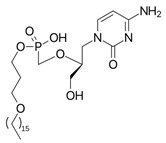	Although Brincidofovir demonstrated encouraging activity in preliminary cell-based studies, its phase II clinical evaluation was halted in 2015 after the manufacturer discontinued its development. Due to the limited number of participants enrolled, the study lacked sufficient data to assess the drug’s clinical effectiveness.	oral formulation; favorable stability profile; limited clinical efficacy data for EVD; favorable manufacturing scalability	[51,53]

**Table 2:** Vaccines under investigation for EBOV prophylaxis.

Vaccine Name	Class and Composition Considerations	Comment	Pharmaceutical and Practical Deployment Considerations	Refs.
cAd3-EBOZ	Replication-defective ChAd3-vectored Ebola virus vaccine encoding EBOV GP, protective in NHPs	The cAd3-EBOZ vaccine showed good tolerability and strong, durable immune responses in Phase I and II trials in U.S., Europe, and Africa. An MVA boost transiently enhanced responses but was not required for sustained antibody levels, supporting use of a single-dose regimen in further trials.	Viral-vector platform; parenteral administration; cold-chain required; manufacturing scalability feasible but dependent on vector production capacity	[68,69]
VesiculoVax	monovalent vaccine consists of a live, attenuated, replication-competent rVSV vector engineered to express the glycoprotein of the EBOV (Kikwit strain).	The VesiculoVax platform has demonstrated good safety and strong immunogenicity in Phase I trials of the monovalent vaccine, inducing high levels of EBOV GP–specific IgG antibodies and balanced cellular immune responses. A tetravalent VesiculoVax vaccine expressing glycoproteins from EBOV, SUDV, MARV, and LASV provided complete protection against lethal challenge in *Cynomolgus macaques*, supporting its further clinical development as a multivalent filovirus and arenavirus vaccine candidate.	Replication-competent viral-vector platform; cold-chain dependent; single-dose deployment advantage; manufacturing scalability not yet established	[70,71,72]
Ebola GP Vaccine	EBOV (Makona strain) GP recombinant nanoparticle vaccine adjuvanted with Matrix-M™	In rhesus monkey studies, the vaccine provided complete protection against EBOV when administered up to three days after infection. In a Phase I clinical trial in adults, the Matrix-M–adjuvanted EBOV GP vaccine was well tolerated and induced strong and durable antibody responses that persisted for at least one year. These findings support continued clinical development of this vaccine platform.	Recombinant protein vaccine with adjuvant; multi-dose regimen; improved formulation flexibility relative to live vectors; manufacturing scalability likely favorable but dependent on adjuvant supply	[73,74]
EBOVΔVP30 whole-virus vaccine	Hydrogen peroxide–inactivated EBOV (Mayinga strain) lacking VP30 for replication deficiency	This vaccine candidate is a non-replicating, genetically modified EBOV (Mayinga strain) that lacks the VP30 gene and is chemically inactivated to ensure safety while preserving immunogenicity. In preclinical studies, two doses of EBOVΔVP30 provided complete protection against lethal challenge in mice, guinea pigs, and *Cynomolgus macaques*, supporting its further evaluation as a promising Ebola vaccine.	Inactivated/replication-deficient whole-virus platform; high manufacturing and containment complexity; manufacturing scalability not yet established	[75]
rERAG333E/ZGP	Replication-Competent Recombinant Rabies Virus Vector Expressing the EBOV GP	The vaccine was tested in dogs, where oral rERAG333E/ZGP immunization induced durable EBOV and rabies virus (RABV) neutralizing antibodies regardless of prior rabies vaccination. The live rERAG333E expressing EBOV GP shows potential as an oral vaccine for free-roaming animals and as a platform for inactivated human vaccines.	Replication-competent viral-vector platform; oral administration potential; manufacturing scalability not yet established	[76]
-	recombinant EBOV encoding a mutant VP35 virus	A recombinant Ebola virus carrying a mutant VP35 protein (VP35m) was developed to restore RIG-I–like receptor signaling and enhance innate immune activation. In non-human primates, VP35m vaccination induced robust immune responses and protective anti-EBOV antibodies, preventing disease following challenge with wild-type Ebola virus. These findings support further investigation of VP35m-based vaccine strategies.	Experimental live recombinant platform; safety, stability, and manufacturing scalability not yet established	[77]
VRC-EBODNA023-00-VP	Comprises two plasmids encoding the wild-type GPs of EBOV and SUDV.	A Phase 1b clinical trial conducted in Uganda showed that the vaccine was well tolerated and induced strong antigen-specific humoral and cellular immune responses. These results supported the development of more advanced Ebola virus vaccines expressing the same wild-type glycoprotein antigens, which were later evaluated during the 2014 West African Ebola outbreak.	DNA vaccine platform; favorable stability profile; no live-virus handling; manufacturing scalability favorable	[78]

**Table 3:** Overview of Dengue Vaccines Under Development.

Vaccine Name	Vaccine Design	General Considerations */Achievements **/Limitations ***	Refs.
TDEN	Tetravalent live-attenuated dengue vaccine produced by PDK serial passage	* Developed through collaboration between the Walter Reed Army Institute of Research (WRAIR) (USA) and GlaxoSmithKline Biologicals (Rixensart, Belgium) as two formulations, F17 and F19 ** Tested in multiple Phase I/II trials; good safety and tolerability; strong tetravalent humoral responses, especially in previously exposed individuals. *** Transient viremia, waning and serotype-imbalanced immunity, limited cellular and durable neutralizing responses; additional studies required to fully assess protective efficacy.	[160,162,163,178,179]
DPIV	Tetravalent, purified, formalin-inactivated dengue vaccine formulated with aluminum hydroxide, AS01E, or AS03B adjuvants	* developed at WRAIR, formulated at 1 or 4 µg per serotype, yielding four formulations and administered as a two-dose regimen one month apart. ** safe and immunogenic in phase I trials; induce tetravalent neutralizing antibodies and durable cellular immunity, including serotype-specific memory B cells and multifunctional CD4^+^ T-cell responses, particularly in previously exposed individuals. *** Neutralizing antibody levels declined rapidly, especially in individuals without prior dengue exposure, raising concerns about long-term protection and ADE risk. Limited efficacy in non-human primate challenge studies and constraints inherent to inactivated vaccines warrant further investigation.	[160,162,163,178,180,181]

* General considerations include development background and formulation details. ** Achievements summarize clinical trial outcomes, including safety, tolerability, and immunogenicity. *** Limitations describe observed constraints, including virological and immunological limitations and remaining efficacy questions.

**Table 5:** Pharmaceutical and formulation characteristics of selected approved therapeutics and vaccines for VHFs.

Product	Platform/Class	Dosage Form	Route of Administration	Formulation and Stability Considerations	Pharmacokinetic and Dosing Characteristics	Storage Requirements
Ansuvimab (Ebanga)	Human monoclonal antibody targeting EBOV glycoprotein	Lyophilized powder for injection	Intravenous infusion	Histidine buffer, sucrose stabilizer, polysorbate-80 surfactant	Single-dose infusion; IgG1 half-life approximately 20–24 days	2–8 °C
Atoltivimab/Maftivimab/Odesivimab (Inmazeb, REGN-EB3)	Triple monoclonal antibody cocktail targeting EBOV glycoprotein	Sterile injectable solution	Intravenous infusion	Histidine buffer system, sucrose stabilizer, polysorbate-80	Single-dose infusion; prolonged IgG circulation (~21–25 days)	2–8 °C
rVSV-ZEBOV (Ervebo)	Live recombinant vesicular stomatitis virus vector expressing EBOV glycoprotein	Frozen liquid suspension	Intramuscular injection	Stabilized viral-vector formulation preserving infectivity	Single-dose vaccine inducing rapid antibody responses	−80 °C to −60 °C for long-term storage; thawed vaccine may be stored at 2–8 °C for a limited period before administration.
Ad26.ZEBOV (Zabdeno)	Recombinant adenovirus type-26 vector vaccine	Liquid suspension	Intramuscular injection	Viral vector formulation with stabilizing buffers and cryoprotectants	Prime dose of heterologous vaccine regimen	−20 °C
MVA-BN-Filo (Mvabea)	Modified vaccinia Ankara viral vector vaccine	Liquid suspension	Intramuscular injection	Stabilized viral vector preparation used as booster in heterologous regimen	Booster dose following Ad26.ZEBOV vaccination	−20 °C
YF-17D (Yellow fever vaccine; e.g., YF-VAX, Stamaril)	Live attenuated flavivirus vaccine	Lyophilized powder requiring reconstitution	Subcutaneous or intramuscular injection	Stabilized live-virus formulation produced using egg-based seed-lot system	Single-dose vaccine providing long-term immunity	2–8 °C
Dengvaxia	Recombinant live attenuated tetravalent dengue vaccine	Lyophilized powder requiring reconstitution	Subcutaneous injection	Stabilized viral vaccine requiring cold-chain maintenance	Three-dose vaccination schedule administered at 0, 6, and 12 months.	2–8 °C

## Data Availability

No new data were created or analyzed in this study.

## Supplementary Materials

The following supporting information can be downloaded at: https://www.mdpi.com/article/10.3390/pharmaceutics18040426/s1, Table S1: Viruses responsible for viral haemorrhagic fevers and their main epidemiological characteristics. Table S2: Major Ebola virus disease outbreaks reported since 1976.

## Abbreviations

The following abbreviations are used in this manuscript:

AHF	Argentine hemorrhagic fever
ALT	Alanine Aminotransferase
AST	Aspartate Aminotransferase
BDBV	Bundibugyo ebolavirus
BSL-4	Biosafety Level 4
BVD	Bundibugyo virus disease
CCHF	Crimean–Congo hemorrhagic fever
CCHFV	CCHFV—Crimean–Congo hemorrhagic fever virus
CDC	US Centers for Disease Control and Prevention
CFDA	China Food and Drug Administration
CG	Republic of the Congo
CI	confidence interval
DENV	Dengue virus
DHF	dengue hemorrhagic fever
DRC	The Democratic Republic of the Congo
EBOD	Ebola disease
EBOV	Ebola virus
EMA	European Medicines Agency
EVD	Ebola virus disease
GP	glycoprotein
HFRS	Hantavirus hemorrhagic fever with renal syndrome
HTNV	Hantaan virus
INRB	Congolese Institut National de Recherche Biomédicale
JUNV	Junin virus
KFD	Kyasanur Forest disease
KFDV	Kyasanur Forest Disease virus
LASV	Lassa virus
LF	Lassa fever
LNP	lipid nanoparticle
mAb	monoclonal antibody
MARV	Marburg virus
mRNA	messenger ribonucleic acid
MVA	multivalent modified vaccinia Ankara
MVD	Marburg virus disease
NHP	nonhuman primate
NIH	U.S. National Institutes of Health
NP	nucleoprotein
OHFV	Omsk hemorrhagic fever virus
RAVN	Ravn virus
RBD	receptor-binding domain
RCT	randomized controlled trial
RNA	ribonucleic acid
RVF	Rift Valley fever
RVFV	Rift Valley Fever virus
SEOV	Seoul hantavirus
siRNA	small interfering ribonucleic acid
SUDV	Sudan virus
SVD	Sudan virus disease
TAFD	Taï Forest Virus Disease
TAFV	Taï Forest virus
UNICEF	United Nations Children’s Fund
UTRs	untranslated regions
VE	vaccine efficacy
VHFs	viral hemorrhagic fevers
VLPs	virus-like particles
VSV	vesicular stomatitis virus
WHO	World Health Organization
WRAIR	Walter Reed Army Institute of Research
YF	Yellow fever
YFV	Yellow fever virus
ZEBOV	Zaire Ebola virus
