# Supplementary material for: Achievements and Challenges in Therapy and Vaccines Development of Viral Hemorrhagic Fevers: An Up-to-Date Review
Source: Pharmaceutics. 2026 Mar 30;18(4):426. doi: 10.3390/pharmaceutics18040426 (PMC13119275; doi:10.3390/pharmaceutics18040426)
Supplement: Supplementary file 1 [file pharmaceutics-18-00426-s001.zip › pharmaceutics-4187967-supplementary.pdf]

Supplementary Material

Table S1. Viruses responsible for viral haemorrhagic fevers and their main epidemiological characteristics.

Virus family	Virus species	Prevalence	Main reservoir of infection	Incubation period (days)
Arenaviridae	*Mammarenavirus lassaense* (Lassa virus)	Benin, Burkina Faso, Ivory Coast, Ghana, Guinea, Liberia, Mali, Nigeria, Sierra Leone, Togo	*Mastomys natalensis* (Multimammate rat)	2-21
	Mammarenavirus lujoense (Lujo virus)	Zambia	It has not yet been identified (probably rodents)	7-11
	Mammarenavirus juninense (Junin virus)	Argentina	*Calomys musculinus* (Drylands vesper mouse)	6-14
	Mammarenavirus chapareense (Chapare virus)	Bolivia	*Oligoryzomys microtis* (Small-eared pygmy rice rats)	4-21
	Mammarenavirus brazilense (Sabia virus)	Brazil	It has not yet been identified (probably rodents)	6-21
	Mammarenavirus machupoense (Machupo virus)	Bolivia	*Calomys callosus* (Large vesper mouse)	3-16
	Mammarenavirus guanaritoense (Guanarito virus)	Venezuela	*Zygodontomys brevicauda* (Short-tailed Cane mouse)	3-19
Filoviridae	*Orthoebolavirus zairense* (Ebola virus, EBOV)	The Democratic Republic of the Congo (DRC), Gabon, Guinea, Republic of the Congo (CG)	probably various species of fruit bats	2-21
	Orthoebolavirus sudanense (Sudan virus, SUDV)	South Sudan, Uganda	probably various species of fruit bats	2-21
	Orthoebolavirus bundibugyoense Bundibugyo ebolavirus (BDBV)	The DRC, Uganda	probably various species of fruit bats	2-21
	Orthoebolavirus taiense (Taï Forest virus, TAFV)	Ivory Coast	probably various species of fruit bats	2-21
	Marburg marburgvirus MARV	Angola, The DRC, Equatorial Guinea, Ghana, Guinea, Kenya, Tanzania, Uganda, Zimbabwe	*Rousettus aegyptiacus* (Egyptian fruit bat)	2-21

Virus family	Virus species	Prevalence	Main reservoir of infection	Incubation period (days)
	Marburg virus<br>Ravn virus,<br>RAVN	The DRC, Kenya, Uganda	Rousettus aegyptiacus<br>(Egyptian fruit bat)	2-21
Flaviviridae	*Orthoflavivirus dengue*<br>(Dengue virus)<br>(DENV)	The Americas, South and Southeast Asia, Western Pacific region, Africa,	mosquitoes of the genus *Aedes*<br>(*Aedes aegypti, Aedes albopictus*)	5-7
	Orthoflavivirus flavi<br>(Yellow Fever virus,<br>YFV)	Tropical areas of Africa and South America	*Aedes aegypti* mosquitoes<br>mosquitoes of the genus *Haemagogus* and *Sabethes*	3-6
	Orthoflavivirus kyasanurense<br>(Kyasanur Forest Disease virus, KFDV)	India	*Hemaphysalis spinigera*<br>(Hard ticks)	3-8
	Orthoflavivirus omskense<br>(Omsk hemorrhagic fever virus, OHFV)	Western Siberia (Omsk Oblast, Novosibirsk Oblast, Kurgan Oblast, Tyumen Oblast)	Ticks from species<br>*Dermacentor reticulatus,*<br>*Dermacentor marginatus,*<br>*Ixodes persulcatus*	3-8
	Alkhurma hemorrhagic fever virus	Saudi Arabia, Egypt, Djibouti, and India	*Ornithodoros savignyi*<br>(soft ticks)<br>*Hyalomma dromedary*<br>(hard ticks)	2-4
Hantaviridae	Several species of *Orthohantavirus*	Europe, America, Asia	rodents	7-56
Nairoviridae	*Orthonairovirus haemorrhagiae*<br>(Crimean-Congo Hemorrhagic Fever virus, CCHFV)	Eastern and southern Europe, Central Asia, Africa and Middle East	*Ixodidae* family<br>(hard ticks)	1-14
Param yxoviridae	*Henipavirus nipahense*<br>(Nipah virus)	Malaysia, Singapore, Bangladesh and India	Megabats from genus *Pteropus*	5-14
	Henipavirus hendraense<br>(Hendra virus)	Australia only	Megabats from genus Pteropus (native to Australia)	9-16
Phenuiviridae	*Phlebovirus riftense*<br>(Rift Valley Fever virus, RVFV)	Eastern and southern Africa	Mosquitos	2-6

Table S2. Major Ebola virus disease outbreaks reported since 1976.

Year	Outbreak cases/deaths/ percent of deaths	Country	Additional cases/ deaths	Country	Comment
1976	318/280/88	The DRC	1/0	United Kingdom	Ebola was first discovered during this outbreak in Équateur Province, mostly within 70 kilometers of Yambuku village. The first patient, thought to have malaria, got an injection at the Yambuku Mission Hospital. The virus spread through reused needles and close contact between people.<br>The UK case happened when a lab worker was accidentally infected by a needle containing the virus.
1977			1/1	The DRC	The case identified in the village of Tandala showed no apparent link to the 1976 Ebola outbreak, indicating that the virus may be naturally maintained within local animal populations in that region.
1994	51/31/61	Gabon			The outbreak emerged in a number of gold-mining settlements located deep within the rainforest near Makakou.
1995	315/254/81	The DRC			The outbreak began in Kikwit and nearby areas, starting with a charcoal worker from the nearby forest. It spread among families and in hospitals, but stopped quickly once healthcare workers began using masks, gloves, and gowns
1996	31/21/68<br>60/45/75	Gabon<br>Gabon			The first outbreak began in spring in Mayibout 2, a village near the Ivindo River, after hunters ate a dead chimpanzee found in the forest. Eighteen people who handled the animal got sick, followed by their family members. The second outbreak occurred in the fall in Booué, starting with a hunter from a logging camp who infected others. Dead chimpanzees in the area tested positive for the Ebola virus.
	-	-	1/1	Russia	A Russian lab worker get infected while testing a possible treatment.
2001	59/44/75<br>65/53/81	CG<br>Gabon			Ebola was first reported in the CG during an outbreak along the Gabon border, affecting Mbomo and Kéllé districts. The first human cases were linked to hunting and contact with wildlife. A second outbreak occurred in the same border region, affecting parts of Gabon, where many dead animals, mainly primates, were found. Like before, the initial human cases were tied to hunting and wildlife contact.
2003	143/128/89<br>35/29/83	CG<br>CG			The first outbreak, from January to April, hit Mbomo and Kéllé districts in western Cuvette Ouest. It started when hunters came into contact with dead or hunted

Year	Outbreak cases/deaths/ percent of deaths	Country	Additional cases/ deaths	Country	Comment
					wildlife, then spread mainly through close contact with infected family members, with little transmission in healthcare settings. The second outbreak, in November-December in Mbomo, involved community awareness efforts, especially targeting women, who are typically the main caregivers.
2004			1/1	Russia	Again, a Russian lab worker get infected while testing a possible treatment.
2005	12/10/83	CG			Most infections occurred among hunters, caregivers, and people attending funerals.
2007	264/187/71	The DRC			The outbreak began in mid-September in Luebo and Mweke health zones, Kasai Occidental Province. Radio broadcasts informed locals about Ebola and how to prevent it. The final confirmed case was on October 4, and the outbreak ended on November 20.
2008	32/15/47	The DRC			The outbreak took place in Mweka and Luebo in Kasai Occidental province, with several international partners helping in the response.
2014	28610/11308/ 39	Guinea, Liberia, Sierra Leone			The West Africa Ebola epidemic, the largest in history, began in southeastern Guinea and was reported by the WHO on March 23, 2014. The virus quickly spread to neighboring Sierra Leone and Liberia. Weak disease surveillance, fragile health systems, and poor infection control made it difficult to contain. In August 2014, the WHO declared it a Public Health Emergency of International Concern. The Centers for Disease Control and Prevention (CDC), alongside international and local partners, worked intensively to control the outbreak, which was officially declared over in June 2016.
	69/49/71	The DRC			This outbreak took place in villages around Boende town in western DRC. The Ebola strain was similar to the one from the 1995 Kikwit outbreak, confirming it was unrelated to the West Africa epidemic occurring at the same time.
			1/0	Italy	An Italian healthcare worker who had served in Sierra Leone during the West Africa Ebola epidemic developed Ebola symptoms three days after returning to Rome.
			8/6	Mali	A traveler carry the Ebola virus from Guinea to Mali.
	20/8/40	Nigeria			During the West Africa Ebola outbreak, a traveler from Liberia brought the virus to Lagos, Nigeria, infecting some responders. Quick action to treat patients, train

Year	Outbreak cases/deaths/ percent of deaths	Country	Additional cases/ deaths	Country	Comment
					staff, and trace contacts kept the outbreak limited to two cities and stopped it from spreading further.
			1/0	Senegal	The Ebola virus reached Senegal through an infected traveler
			1/0	Spain	During the West Africa Ebola outbreak, a Spanish healthcare worker became infected while caring for a patient evacuated from Sierra Leone, marking the first human-to-human Ebola transmission outside Africa.
			1/0	UK	During the West Africa Ebola outbreak, a healthcare worker developed symptoms after returning to the UK from volunteering at a treatment center in Sierra Leone.
			4/1	USA	During the West Africa Ebola outbreak, 4 cases (1 death) were reported in the United States. In addition, several Ebola patients infected abroad were medically evacuated to the U.S. for treatment, resulting in a total of 11 patients treated in U.S. healthcare facilities during the outbreak.
2017	8/4/50	The DRC			The outbreak began in Bas Uélé province. The Ministry of Public Health, supported by CDC, WHO and other partners, led efforts in detection, treatment, and communication. Response teams faced major challenges due to the area's remoteness and limited infrastructure.
2018	54/33/61.1	The DRC			The ninth outbreak of the DRC began in May in the Bikoro region and ends on the late July
	3470/2287/66	The DRC			The tenth outbreak of the DRC began in August in North Kivu province and it lasted almost 2 years.
2020	130/55/42.3	The DRC			In June, the DRC announced its 11th Ebola outbreak in Mbandaka, western DRC, separate from the ongoing 10th outbreak in the east. Some cases were likely connected to the 2018 outbreak, probably through relapse or sexual transmission. The outbreak ended in November.
2021	12/6/50	The DRC			The outbreak began in North Kivu Province, which had also been hit by Ebola during the 2018–2020 epidemic, the largest in DRC's history. Genetic testing showed the new cases were connected to that earlier outbreak, likely caused by a survivor's relapse or sexual transmission. It was declared over in May.
	23/12/52.2	Guinea			Ebola cases were confirmed in N'Zérékoré Prefecture in southeastern Guinea. Genetic testing showed the virus matched strains from the 2014–2016 West Africa outbreak, suggesting it likely came from a survivor's

Year	Outbreak cases/deaths/ percent of deaths	Country	Additional cases/ deaths	Country	Comment
					lingering infection rather than a new spillover from animals.
	11/9/82	The DRC			In October, an Ebola outbreak was reported in North Kivu Province. Some earlier cases from September were later confirmed. Tests linked the virus to the 2018–2020 outbreak, likely from a survivor's relapse. It was the DRC's 13th Ebola outbreak and ended in December.
2022	5/5/100	The DRC			The 14th Ebola outbreak in DRC (April-July)
	1/1/100	The DRC			The 15th Ebola outbreak in DRC (August-September)
2025	64/45/70.3	The DRC			On September 4, 2025, an Ebola outbreak was declared in Bulape, Kasai Province. By mid-October, 64 cases and 45 deaths had been reported, and over 32,000 people were vaccinated. This marks the DRC's 16th Ebola outbreak since 1976.
